# Association of Intraoperative Transesophageal Echocardiography and Clinical Outcomes After Open Cardiac Valve or Proximal Aortic Surgery

**DOI:** 10.1001/jamanetworkopen.2021.47820

**Published:** 2022-02-09

**Authors:** Emily J. MacKay, Bo Zhang, John G. Augoustides, Peter W. Groeneveld, Nimesh D. Desai

**Affiliations:** 1 Department of Anesthesiology and Critical Care, Perelman School of Medicine at the University of Pennsylvania, Philadelphia; 2 Penn Center for Perioperative Outcomes Research and Transformation (CPORT), University of Pennsylvania, Philadelphia; 3 Penn’s Cardiovascular Outcomes, Quality and Evaluative Research Center (CAVOQER), University of Pennsylvania, Philadelphia; 4 Leonard Davis Institute of Health Economics (LDI), University of Pennsylvania, Philadelphia; 5 Department of Statistics, The Wharton School, University of Pennsylvania, Philadelphia; 6 Department of Internal Medicine, Perelman School of Medicine at the University of Pennsylvania, Philadelphia; 7 Corporal Michael J. Crescenz Veterans Affairs Medical Center, Philadelphia; 8 Division of Cardiovascular Surgery, Perelman School of Medicine at the University of Pennsylvania, Philadelphia

## Abstract

**Importance:**

Intraoperative transesophageal echocardiography (TEE) is used frequently in cardiac valve and proximal aortic surgical procedures, but there is a lack of evidence associating TEE use with improved clinical outcomes.

**Objective:**

To test the association between intraoperative TEE use and clinical outcomes following cardiac valve or proximal aortic surgery.

**Design, Setting, and Participants:**

This matched, retrospective cohort study used national registry data from the Society of Thoracic Surgeon (STS) Adult Cardiac Surgery Database (ACSD) to compare clinical outcomes among patients undergoing cardiac valve or proximal aortic surgery with vs without intraoperative TEE. Statistical analyses used optimal matching within propensity score calipers to conduct multiple matched comparisons including within-hospital and within-surgeon matches, a negative control outcome analysis, and sensitivity analyses. STS ACSD data encompasses more than 90% of all hospitals that perform cardiac surgery in the US. The study cohort consisted of all patients aged at least 18 years undergoing open cardiac valve repair or replacement surgery and/or proximal aortic surgery between 2011 and 2019. Statistical analysis was performed from October 2020 to April 2021.

**Exposures:**

The exposure was receipt of intraoperative TEE during the cardiac valve or proximal aortic surgery.

**Main Outcomes and Measures:**

The primary outcome was death within 30 days of surgery. The secondary outcomes were (1) a composite outcome of stroke or 30-day mortality and (2) a composite outcome of reoperation or 30-day mortality.

**Results:**

Of the 872 936 patients undergoing valve or aortic surgery, 540 229 (61.89%) were male; 63 565 (7.28%) were Black and 742 384 (85.04%) were White; 711 326 (81.5%) received TEE and 161 610 (18.5%) did not receive TEE; the mean (SD) age was 65.61 years (13.17) years. After matching, intraoperative TEE was significantly associated with a lower 30-day mortality rate compared with no TEE: 3.81% vs 5.27% (odds ratio [OR], 0.69 [95% CI, 0.67-0.72]; *P* < .001), a lower incidence of stroke or 30-day mortality: 5.56% vs 7.01% (OR, 0.77 [95% CI, 0.74-0.79]; *P* < .001), and a lower incidence of reoperation or 30-day mortality: 7.18% vs 8.87% (OR, 0.78 [95% CI, 0.76-0.80]; *P* < .001). Results were similar across all matched comparisons (including within-hospital, within-surgeon matched analyses) and were robust to a negative control and sensitivity analyses.

**Conclusions and Relevance:**

Among adults undergoing cardiac valve or proximal aortic surgery, intraoperative TEE use was associated with improved clinical outcomes in this cohort study. These findings support routine use of TEE in these procedures.

## Key Points

**Question:**

Is intraoperative transesophageal echocardiography (TEE) use associated with improved clinical outcomes among patients undergoing cardiac valve or proximal aortic surgery?

**Findings:**

This matched cohort study of 872 936 patients undergoing cardiac valve or aortic surgery between 2011 and 2019 found that intraoperative TEE use was associated with lower 30-day mortality, a lower incidence of stroke or 30-day mortality, and a lower incidence of cardiac reoperation or 30-day mortality.

**Meaning:**

These findings suggest that intraoperative TEE may improve clinical outcomes after open cardiac valve (repair or replacement) and/or aortic surgery.

## Teaser

This cohort study examines the association between intraoperative transesophageal echocardiography (TEE) use and clinical outcomes following cardiac valve or proximal aortic surgery among US adults.

## Introduction

Each year, 150 000 patients undergo high-risk,^1,2,3^ open cardiac valve or proximal aortic surgery in the US.^4^ Transesophageal echocardiography (TEE) is an ultrasonography-based, cardiac imaging tool used in cardiac surgical procedures to facilitate informed surgical decision making^5,6,7^ and manage intraoperative complications.^5,6,7,8,9^ However, the current American Heart Association (AHA) and American College of Cardiology (ACC) guidelines do not specifically recommend for or against the use of intraoperative TEE in the majority of cardiac surgical procedures^10,11,12^ because prior to 2020, observational studies have not directly associated intraoperative TEE with improved clinical outcomes.^5,6,7,8,9^ Recently, evidence has begun to accumulate on improved outcomes with TEE use after cardiac valve and coronary artery bypass graft (CABG) surgical procedures.^13,14,15^ But there is no research directly comparing outcomes after proximal aortic surgery with TEE vs without TEE, and the existing study on improved outcomes with TEE after cardiac valve repair or replacement surgery used administrative claims data.^15^

To go beyond prior observational work using claims data,^15,16^ this study aimed to test the association between intraoperative TEE and clinical outcomes using data from the Society of Thoracic Surgeon (STS), Adult Cardiac Surgery Data (ACSD) registry database. These STS data allowed the application of rigorous statistical matching techniques to directly compare similar patients who underwent cardiac valve or proximal aortic surgery with vs without intraoperative TEE. We hypothesized that intraoperative TEE would be associated with a decreased incidence of 30-day mortality, stroke or 30-day mortality, and reoperation or 30-day mortality.

## Methods

### Data Sources

The STS ACSD contains 6.9 million surgical records, has 3800 participating surgeons, and encompasses more than 90% of the hospitals that perform cardiac surgery in the US.^17^ For this analysis, data across STS ACSD versions 2.73, 2.81, and 2.90 were queried.^18^ All data management and statistical analyses were performed in accordance with the STS Participant User Files Data Use Agreement. Our study adheres to the Strengthening the Reporting of Observational Studies in Epidemiology (STROBE) reporting guideline for observational studies.^19^ All aspects of this study were reviewed and approved by the University of Pennsylvania institutional review board and informed consent was waived given the deidentified nature of the data.

This study’s data (including race and ethnicity) were collected as variables retrospectively by each institution participating in the STS ACSD. Because this study analyzed national data across multiple institutions, it is unknown how each institution recorded race and ethnicity (eg, whether by electronic medical record or by patient report). However, the STS Research Center quality-controls all variables by audit regularly.

### Population

The study cohort consisted of all patients aged at least 18 years undergoing at least one of the following surgical procedures between July 1, 2011, and June 30, 2019: (1) open valve (aortic, mitral, pulmonic, or tricuspid) repair or replacement; (2) open, ascending aortic, and/or proximal aortic arch surgery (eg, aortic root replacement with a valved conduit, aortic valve sparing root, aortic homograft, or nonvalved conduit replacement with or without aortic hemiarch replacement). Patients were excluded if undergoing any of the following surgical procedures: (1) isolated CABG surgery; (2) isolated other cardiac surgery; (3) unspecified valve repair or replacement surgery; or (4) unspecified aortic surgery.

### Outcomes

Our primary outcome was death within 30 days of surgery.^20^ Our secondary outcomes were (1) composite of in-hospital stroke or 30-day mortality; or (2) composite of in-hospital reoperation (for bleeding, valve or CABG reintervention) or 30-day mortality. Information on outcome variable labels across STS ACSD versions (eg, 2.73, 2.81, and 2.90) may be found in eAppendix 2 in the Supplement.

### Exposure

The exposure variable was receipt of an intraoperative TEE. This was defined using the STS ACSD variable called intraoperative TEE post procedure (consistent across versions 2.73, 2.81, and 2.90).

### Key Covariates

Independent covariates were used for matching. The categories included: demographics, admission status, preexisting comorbidities, hemodynamic data, laboratory values, intraoperative surgical variables, surgery type, surgical volume by hospital and surgeon and STS projected risk scores.

### Statistical Matching

Because of baseline covariate differences (eAppendix 1 in the Supplement) between patients undergoing cardiac valve or proximal aortic surgery with vs without intraoperative TEE, we performed 2 matched comparisons.^21,22^ First, an all-patient, across-hospital, across-surgeon matched comparison and a second, within-hospital, within-surgeon matched comparison. All matched analyses involved exact matching on key covariates, finely balancing the joint distribution of key nominal variables,^23^ and balanced on all remaining variables. The all-patient matched comparison was based on optimal matching within propensity score caliper. The 2 within-surgeon matched comparisons were based on optimal subset matching. A detailed discussion on statistical matching methodology is presented in eAppendix 4 in the Supplement.

### All-Patient Matched Comparison

In the first all-patient, across-hospital, across-surgeon, matched comparison, each patient who did not receive a TEE was matched to a comparable patient who did receive TEE during surgery. To ensure each matched pair of patients were as similar as possible, we applied strict matching criteria. First, we matched exactly on New York Heart Association (NYHA) Classification (1 to 4 or absent) and projected 30-day mortality by quartile. Next, because TEE differed across surgery types (eAppendix 1 in the Supplement), we finely balanced^23^ on the 9 major surgery types, secondary procedures, an indicator of previous cardiac surgery, (ie, redo sternotomy) normal ejection fraction (eg, EF greater than or equal to 55%), preexisting hypertension, and admission status. Finally, we balanced all other variables under the categories of demographics, preexisting conditions, hemodynamic data, laboratory values, intraoperative surgical variables, cardiac surgical volume (by hospital and surgeon), and surgery type. Optimal matching within propensity score calipers was implemented using the R package bigmatch. See eAppendix 4 in the Supplement for details on the overall statistical matching methodology and for the all-patient matched comparison.

### Within-Hospital, Within-Surgeon, Matched Comparison

**Figure 1.**
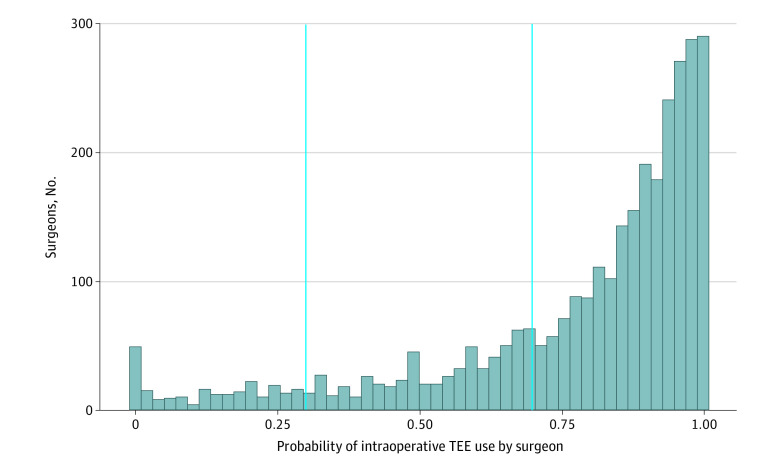
Surgeons’ Overall Preference for TEE This histogram plots the overall distribution of surgeons’ preference (probability intraoperative TEE use by surgeon). The blue lines demarcate surgeons with equivocal preference for intraoperative TEE (eg, surgeons with probability of TEE use between 0.30 and 0.70).

Because there were differences in TEE by hospital and by surgeon (eAppendix 3 in the Supplement), we elected to undertake a second, within-hospital, within-surgeon, matched comparison. For this analysis, each patient undergoing a valve or aortic surgery at a given hospital, by a given surgeon with TEE, was matched to a similar patient undergoing valve or aortic surgery at that same hospital by the same surgeon without TEE. Because intraoperative TEE varied predominantly by surgery type (eAppendix 1 in the Supplement), we applied exact matching to all 9 surgical categories. Covariates used for exact matching included (1) hospital; (2) surgeon; (3) all 9 surgery types; (4) normal EF ( at least 55%); (5) NYHA Classification; and (6) projected 30-day mortality (by quartile). As was done in the all-patient match, we balanced all other variables as specified above. To further reduce selection bias that could occur with surgeons who always (or never) used TEE during cardiac surgery, we considered only surgeons whose preference for TEE was equivocal (TEE probability range of 0.30 to 0.70) (Figure 1). Finally, we elected to conduct an additional, supplementary, within-hospital, within-surgeon matched comparison across all surgeons, regardless of intraoperative TEE frequency (TEE probability range 0.00 to 1.00). To characterize differences in outcomes by surgery type (with TEE vs without), we performed subgroup analyses among patients undergoing similar surgical procedures. These subgroups were categorized based on anatomical location of surgery, surgery type, and those with similar risk profiles. The surgical procedures classified into each subgroup may be found in eAppendix 7 in the Supplement. Statistical matching was implemented using the R package rcbsubset with default settings.

### Statistical Analysis

The quality of statistical matching was assessed using standardized differences (SD). A match was considered acceptable if all covariates had a SD less than 0.10 between the TEE and the no-TEE groups.^21,24,25^

We first conducted an unmatched, unadjusted, analysis of outcomes, where patients undergoing cardiac valve or aortic surgery with vs without TEE were compared. We analyzed the binary clinical outcomes using the Fisher exact test. We next conducted an analysis of outcomes among the matched cohorts. The binary clinical outcomes were analyzed using the McNemar test.^26,27^

Statistical sensitivity analyses were conducted to assess the robustness of our findings to unmeasured confounding using Rosenbaum bounds and amplification techniques.^27,28^ The sensitivity analysis excluding those missing the TEE exposure was conducted using the same statistical tests as previously described. The negative control outcome analysis of elevation in postoperative creatinine was analyzed using a *t* test (unadjusted) and a difference-in-means estimator for the matched pair study design.^29^ All hypothesis testing was 2-sided and significance was set at *P* < .05. Data management, including data cleaning, data categorizing, and merging across ACSD versions was done using Stata version 15.0 (StataCorp). Additional data management required for matching and statistical analyses were conducted by R version 4.0.3 (R Project for Statistical Computing) using the R package dplyr.^30,31^ Statistical analysis was performed from October 2020 to April 2021. A link to the GitHub code repository is provided in eAppendix 11 in the Supplement.

## Results

### Unadjusted, Unmatched Analysis

**Figure 2.**
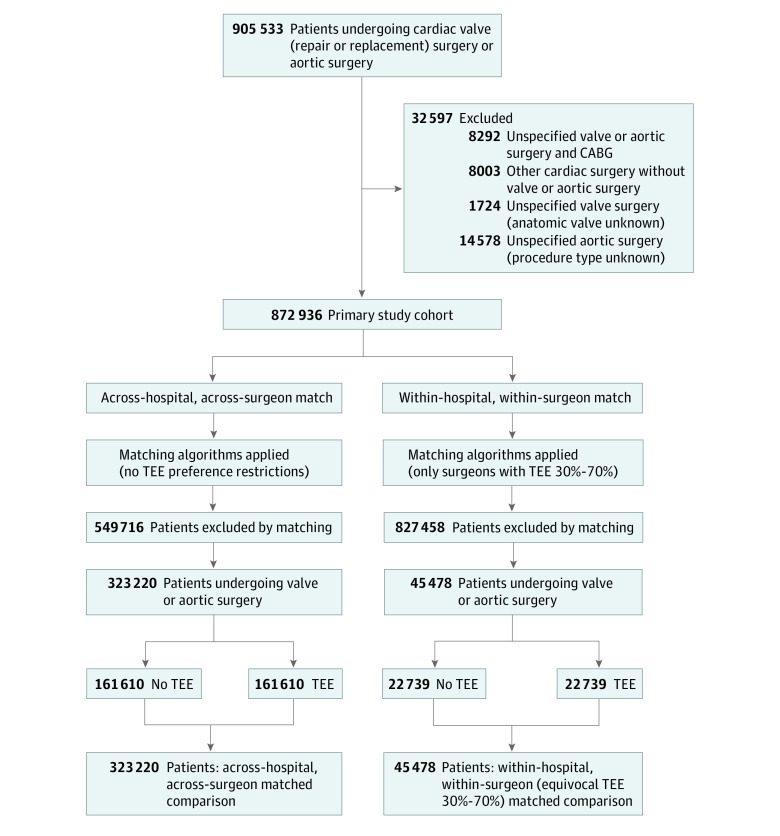
Study Participant Flow Diagram Overview of the study design. The left panels illustrate the all-patient, across-hospital, across-surgeon matched comparison. The right panels illustrate the within-hospital, within-surgeon (with equivocal TEE preference: TEE 0.30–0.70) matched comparison.

Following exclusions (Figure 2), our study cohort included 872 936 patients undergoing valve or aortic surgery. Of the 872 936 patients, 540 229 (61.89%) were male, 63 565 (7.28%) were Black, 742 384 (85.04%) were White, 711 326 (81.5%) received TEE, and 161 610 (18.5%) did not receive TEE; the mean (SD) age was 65.61 years (13.17) years. Compared with patients who did not receive TEE, those who did receive TEE were similar demographically and hemodynamically, but had higher rates of preexisting comorbidities, (Table 1) and varied by surgery type. The complete baseline characteristics between the TEE vs no TEE groups and the TEE distribution by surgery type are presented in eAppendix 1 in the Supplement.

**Table 1.** Baseline Characteristics of the Study Cohort

Characteristic^a^^,^^b^	No. (%)		Standardized mean difference
	TEE (n = 711 326)	No TEE (n = 161 610)	
Demographics and admission type			
Age, mean (SD), y	65.49 (13.17)	66.14 (13.17)	−0.050
BMI, mean (SD)^c^	29.29 (10.44)	29.47 (11.04)	−0.017
Male	440 033 (61.86)	100 196 (62.00)	−0.003
Female	271 088 (38.11)	61 355 (37.96)	
Black	52 062 (7.32)	11 503 (7.12)	0.008
White	603 895 (84.90)	138 489 (85.69)	−0.022
Elective admission	532 262 (74.83)	119 996 (74.25)	0.013
Preexisting comorbid conditions			
NYHA class			
1	18 357 (2.58)	3396 (2.10)	0.031
2	89 665 (12.61)	18 325 (11.34)	0.039
3	132 911 (18.68)	28 116 (17.40)	0.033
4	60 965 (8.57)	12 894 (7.98)	0.021
Infectious endocarditis	59 482 (8.36)	12 342 (7.64)	0.026
Home oxygen	19 763 (2.78)	3841 (2.38)	0.025
Sleep apnea	105 016 (14.76)	20 032 (12.40)	0.068
Liver diseases	33 762 (4.75)	6215 (3.85)	0.043
Cancer	37 811 (5.32)	7852 (4.86)	0.021
CVD	123 290 (17.33)	26 435 (16.36)	0.026
CVA	60 958 (8.57)	13 275 (8.21)	0.013
Hemodynamic data & laboratory values, mean (SD)			
PASYS, mm Hg	40.89 (11.81)	40.74 (10.57)	0.013
Ejection fraction, %	55.24 (11.90)	55.16 (11.95)	0.007
Creatinine, mg/dL	1.18 (1.02)	1.19 (1.00)	−0.005
Albumin, g/dL	3.79 (0.57)	3.77 (0.56)	−0.070
Surgical variables & intraoperative data			
Previous CABG surgery	36 607 (5.15)	9279 (5.74)	−0.027
Previous valve surgery	66 877 (9.40)	14 360 (8.89)	0.018
CPB time, mean (SD), min	138.45 (64.03)	140.38 (68.15)	−0.030
Time in OR, mean (SD), min	356.40 (117.72)	357.43 (123.44)	−0.009
Surgery type			
Aortic valve			
Repair	11 625 (1.63)	2221 (1.37)	0.021
Replacement	412 751 (58.03)	101 961 (63.09)	−0.103
Mitral valve			
Repair	154 423 (21.71)	25 546 (15.81)	0.146
Replacement	126 991 (17.85)	28 191 (17.44)	0.011
Tricuspid valve repair/replacement	67 817 (9.53)	11 603 (7.18)	0.082
Pulmonic valve repair/replacement	3914 (0.55)	876 (0.54)	0.001
Aortic root/valve conduit (Bentall)	30 715 (4.32)	6943 (4.30)	0.001
Aortic valve sparing root	29 946 (4.21)	5623 (3.48)	0.037
Aortic homograft or non-valved conduit	10 926 (1.54)	2858 (1.77)	−0.019
Plus CABG	217 360 (30.56)	53 631 (33.19)	−0.057
Plus other cardiac surgery	204 702 (28.78)	40 471 (25.04)	0.083
Cumulative valve/aortic surgery volume, mean (SD)			
Hospital-level	2442.03 (3497.25)	2030.32 (2916.90)	0.121
Surgeon-level	667.31 (566.87)	567.29 (476.10)	0.181
STS mortality risk projection score			
Projected mortality, mean (SD), %	3.63 (3.92)	3.77 (4.17)	−0.103

Abbreviations: BMI, body mass index; CABG, coronary artery bypass graft; CPB, cardiopulmonary bypass time; CVA, cerebrovascular accident; CVD, cerebrovascular disease; NYHA, New York Heart Association; OR, operating room; PASYS, pulmonary arterial systolic; TEE, transesophageal echocardiography.

SI conversion factors: To convert creatinine to micromoles per liter, multiply by 88.4; to convert albumin to grams per liter, multiply by 10.

^a^ Mean and standard deviation are reported for continuous variables. Counts and percentages are reported for binary, categorical, and ordinal variables.

^b^ Selected covariates are presented. The full baseline characteristics table is presented in eAppendix 1 in the Supplement.

^c^ BMI is calculated as weight in kilograms divided by height in meters squared.

Overall, 39 078 patients (4.32%) died within 30 days. Patients who received an intraoperative TEE had a lower 30-day mortality: 3.92% vs 5.27% (odds ratio [OR], 0.73 [95% CI, 0.72-0.75]; *P* < .001), a lower incidence of stroke or 30-day mortality: 5.63% vs 7.01% (OR, 0.79 [95% CI, 0.77-0.81]; *P* < .001), and a lower incidence of reoperation or 30-day mortality: 7.31% vs 8.87% (OR, 0.81 [95% CI, 0.79-0.83]; *P* < .001). Unadjusted outcomes reported in McNemar format may be found in eAppendix 6 in the Supplement.

### Matching Assessment

Our first, across-hospital, across-surgeon match consisted of 161 610 matched pairs that were similar in observable covariates (Table 2). After matching, standardized differences across all variables were less than 0.10. The full covariate balance after matching is presented in eAppendix 5 in the Supplement. Our second, within-hospital, within equivocal-TEE-surgeon match consisted of 22 739 matched pairs that were similar in observable covariates; all with standardized differences less than 0.10. The full covariate balance is presented in eAppendix 5 in the Supplement.

**Table 2.** Covariate Balance After the All-Patient, Across-Hospital, Across-Surgeon Match

Covariate^a^^,^^b^	No. (%)		Standardized difference
	TEE = 0 (n = 161 610)	TEE = 1 (n = 161 610)	
Demographics and admission type			
Age, mean (SD), y	66.14 (13.17)	66.32 (13.01)	0.014
BMI, mean (SD)^c^	29.47 (11.04)	29.16 (8.19)	−0.030
Male	100 196 (62.00)	103 109 (63.80)	0.037
Female	61 414 (38.00)	58 501 (36.20)	
Black	11 503 (7.12)	11 427 (7.07)	−0.002
White	138 489 (85.69)	138 678 (85.81)	0.003
Elective admission	119 996 (74.25)	120 027 (74.27)	0.000
Preexisting comorbid conditions			
NYHA class^c^			
1	3396 (2.10)	3396 (2.10)	0
2	18 325 (11.34)	18 325 (11.34)	0
3	28 116 (17.40)	28 116 (17.40)	0
4	12 894 (7.98)	12 894 (7.98)	0
Infectious endocarditis	12 342 (7.64)	12 092 (7.48)	−0.006
Home oxygen	3841 (2.38)	2765 (1.71)	−0.041
Sleep apnea	20 032 (12.40)	21 413 (13.25)	0.024
Liver diseases	6215 (3.85)	5056 (3.13)	−0.034
Cancer	7852 (4.86)	6646 (4.11)	−0.034
CVD	26 435 (16.36)	27 383 (16.94)	0.015
CVA	13 275 (8.21)	14 799 (9.16)	0.034
Hemodynamic data & laboratory values, mean (SD)			
PASYS, mm Hg	40.74 (10.57)	40.62 (10.08)	−0.011
Ejection fraction, %	55.16 (11.95)	54.95 (11.56)	−0.018
EF normal	98 157 (60.74)	98 242 (60.79)	0.001
Creatinine, mg/dL	1.19 (1.00)	1.18 (0.98)	−0.011
Albumin, g/dL	3.77 (0.56)	3.77 (0.53)	0.007
Surgical variables & intraoperative data			
Previous CABG surgery	9279 (5.74)	6887 (4.26)	−0.066
Previous valve surgery	14 360 (8.89)	14 128 (8.74)	−0.005
CPB time, mean (SD), min	140.38 (68.15)	139.81 (63.14)	−0.009
Time in OR, mean (SD), min	357.43 (123.44)	353.28 (114.23)	−0.035
Surgery type			
Aortic valve			
Repair	2221 (1.37)	2265 (1.40)	0.002
Replacement	101 961 (63.09)	101 952 (63.09)	−0.000
Mitral valve			
Repair	25 546 (15.81)	25 548 (15.81)	−0.000
Replacement	28 191 (17.44)	28 175 (17.43)	−0.000
Tricuspid valve repair/replacement	11 603 (7.18)	9881 (6.11)	−0.037
Pulmonic valve repair/replacement	876 (0.54)	563 (0.35)	−0.026
Aortic root/valve conduit (Bentall)	6943 (4.30)	7182 (4.44)	0.008
Aortic valve sparing root	5623 (3.48)	5406 (3.35)	−0.007
Aortic homograft or non-valved conduit	2858 (1.77)	2452 (1.52)	−0.020
Plus CABG	53 631 (33.19)	53 500 (33.10)	−0.002
Plus other cardiac surgery	40 471 (25.04)	40 452 (25.03)	−0.000
Cumulative valve/aortic surgery volume, mean (SD)			
Hospital-level	2030.32 (2916.90)	1925.52 (2628.68)	−0.031
Surgeon-level	567.29 (476.10)	546.58 (439.14)	−0.037
STS mortality risk projection score			
Projected mortality quartile^d^			
NA	52 411 (32.43)	52 411 (32.43)	0
1	15 626 (9.67)	15 626 (9.67)	0
2	23 780 (14.71)	23 780 (14.71)	0
3	33 131 (20.50)	33 131 (20.50)	0
4	36 662 (22.69)	36 662 (22.69)	0
STS mortality projection score, mean (SD), %	3.77 (4.17)	3.73 (3.82)	−0.004

Abbreviations: BMI, body mass index; CABG, coronary artery bypass graft; CPB, cardiopulmonary bypass time; CVA, cerebrovascular accident; CVD, cerebrovascular disease; NYHA, New York Heart Association; OR, operating room; PASYS, pulmonary arterial systolic; STS, Society of Thoracic Surgeons; TEE, transesophageal echocardiography.

SI conversion factors: To convert creatinine levels to micromoles per liter, multiply by 88.4; to convert albumin to grams per liter, multiply by 10.

^a^ Mean and standard deviation are reported for continuous variables. Counts and percentages are reported for binary, categorical, and ordinal variables.

^b^ Selected covariates are presented. The full baseline characteristics table is presented in the eAppendix 5 in the Supplement.

^c^ BMI is calculated as weight in kilograms divided by height in meters squared.

^d^ Indicates covariate used for exact matching.

### All-Patient, Across-Hospital, Across-Surgeon Matched Comparison

The all patient across-hospital, across-surgeon matched analysis found that that among 161 610 matched pairs, intraoperative TEE was significantly associated with a lower 30-day mortality rate: 3.81% vs 5.27% (OR, 0.69 [95% CI, 0.67-0.72]; *P* < .001), a lower incidence of stroke or 30-day mortality: 5.56% vs 7.01% (OR, 0.77 [95% CI, 0.74-0.79]; *P* < .001), and a lower incidence of reoperation or 30-day mortality: 7.18% vs 8.87% (OR, 0.78 [95% CI, 0.76-0.80]; *P* < .001) (Table 3). Outcomes reported in McNemar format may be found in eAppendix 6 in the Supplement.

**Table 3.** Study Outcomes by Analysis

Outcomes	Patients, %		OR (95% CI)	*P* value
	TEE	No TEE		
Unadjusted, unmatched comparison				
No.	711 326	161 610		
30-d				
Mortality	3.92	5.27	0.73 (0.72-0.75)	<.001
Mortality or stroke	5.63	7.01	0.79 (0.77-0.81)	<.001
Mortality or reoperation	7.31	8.87	0.81 (0.79-0.83)	<.001
All-patient, across-hospital, across-surgeon matched comparison				
No.	161 610	161 610		
30-d				
Mortality	3.81	5.27	0.69 (0.67-0.72)	<.001
Mortality or stroke	5.56	7.01	0.77 (0.74-0.79)	<.001
Mortality or reoperation	7.18	8.87	0.78 (0.76-0.80)	<.001
Within hospital, within surgeon (equivocal TEE preference: 30%-70%)^a^^,^^b^				
No.	22 739	22 739		
30-d				
Mortality	2.79	3.22	0.86 (0.77-0.96)	.008
Mortality or stroke	4.38	4.76	0.91 (0.83-1.00)	.048
Mortality or reoperation	6.04	6.24	0.94 (0.85-1.04)	.24

Abbreviations: OR, odds ratio; TEE, transesophageal echocardiography.

^a^ Results of a supplementary, within hospital, within surgeon (regardless of TEE preference) is presented in eTable 20 in the Supplement.

^b^ Results of subgroup analyses comparing TEE vs no TEE within similar surgical subgroups is presented in eTable 21 and eTable 22 in the Supplement.

### Within-Hospital, Within-Surgeon Matched Comparison

The within-hospital, within-surgeon with equivocal TEE preference (TEE probability: 0.30-0.70), matched analysis found that among 22 739 matched pairs, intraoperative TEE was significantly associated with a lower 30-day mortality rate: 2.79% vs 3.22% (OR, 0.86 [95% CI, 0.77-0.96]; *P* = .008) and a lower incidence of stroke or 30-day mortality: 4.38% vs 4.76% (OR, 0.91 [95% CI, 0.83-1.00]; *P* = .048). Intraoperative TEE was not statistically significantly associated with a lower incidence of reoperation or 30-day mortality: 5.58% vs 5.77% (OR, 0.94 [95% CI, 0.85-1.04]; *P* = .24) (Table 3). The 30-day mortality on the within-hospital, within-surgeon matched comparison was approximately 1% to 2% lower than the all-patient, across-hospital, across-surgeon matched comparison (30-day mortality with TEE: 3.81% on all-patient, across-hospital, across-surgeon matched comparison vs 2.79% on the within-hospital, within-surgeon, matched comparison; 30-day mortality without TEE: 5.27% on all-patient, across-hospital, across-surgeon matched comparison vs 3.22% on the within-hospital, within-surgeon, matched comparison) (Table 3). Outcomes reported in McNemar format may be found in eAppendix 6 in the Supplement. An additional supplementary within-hospital, within-surgeon matched comparison across all surgeons, regardless of the probability of intraoperative TEE use (65 340 matched pairs), found comparable results (eAppendix 6 in the Supplement). Additional subgroup analyses investigating outcomes with TEE vs without among patients indicated that patients undergoing mitral valve replacement or proximal aortic surgical procedures seem to benefit more from TEE compared with the overall cohort. These results are presented in eAppendix 7 in the Supplement.

### Sensitivity Analyses and Negative Control Analysis

To test the robustness of our findings, we completed sensitivity analyses and a negative control outcome analysis. The first sensitivity analysis indicated that according to the Rosenbaum bounds and associated amplification analysis,^27,28^ to nullify the primary outcome finding from the all-patient, across-hospital, across-surgeon matched comparison, it would take an unmeasured confounder that doubled the odds of 30-day mortality and tripled the odds of TEE use (eAppendix 8 in the Supplement). To nullify the primary outcome finding from the within-hospital, within-surgeon matched comparison, it would take an unmeasured confounder that increased the odds of 30-day mortality by 40% and the odds of TEE use by more than 40% (eAppendix 8 in the Supplement). The second sensitivity analysis tested the robustness of our results by excluding the 2% of the cohort missing the TEE exposure and revealed findings that agreed with our presented results (eAppendix 9 in the Supplement). Finally, our negative control outcome analysis compared elevation in postoperative creatinine between the TEE and no-TEE groups. Three of the 4 negative control outcome analyses were either statistically insignificant or incongruent with the primary results—an additional indication that residual confounding was controlled (eAppendix 6 in the Supplement). A detailed explanation of the negative control outcome including rationale for selection and interpretation of the results is presented in eAppendix in the Supplement.

## Discussion

Among 872 936 patients undergoing valve or aortic surgery, across all analyses, intraoperative TEE was statistically significantly associated with a lower 30-day mortality, a lower incidence of stroke or 30-day mortality, and in the all-patient match, TEE was statistically significantly associated with a lower incidence of reoperation or 30-day mortality. These results were supported by multiple sensitivity analyses^27,28^ that established the presented results would remain statistically significant at a .05 level in the presence of an unmeasured confounder that doubled the odds of 30-day mortality and tripled the odds of intraoperative TEE use, suggesting the presented findings would be robust to residual, unmeasured confounding.^22,28^

Current AHA/ACC guidelines^10,11^ do not specifically recommend for or against the use of intraoperative TEE for all cardiac valve replacement surgical procedures,^10^ most cardiac valve repair surgical procedures,^10^ and all proximal aortic aneurysm surgical procedures.^11^ Presumably, this equivocal, class IIa, AHA/ACC stance on intraoperative TEE use is due to the absence of research comparing clinical outcomes among patients undergoing cardiac surgery with vs without TEE.^5,6,8,9^ Only very recently has the impact of intraoperative TEE on clinical outcomes among patients undergoing cardiac surgery with vs without TEE been directly compared.^14,15,16^

The current study’s finding that intraoperative TEE is associated with improved clinical outcomes is consistent with recent previous comparative effectiveness research by both our group^15,16^ and others.^14^ In 2020, we used propensity score matching to compare 219 238 Medicare beneficiaries undergoing cardiac valve surgery and found TEE was associated with a lower 30-day mortality.^15^ In 2021, we used instrumental variable methods to compare 114 871 Medicare beneficiaries undergoing isolated CABG surgery and found that TEE was associated with lower in-hospital stroke and lower 30-day mortality.^13^ Subsequently, an independent study by Metkus and colleagues used STS data and propensity score matching to compare 1.3 million patients undergoing isolated CABG surgery with vs without TEE and found a mortality benefit to the use of TEE.^14^

The current study improves upon our previous work^15,16^ in several noteworthy respects. First, the detailed, patient-level data found in the STS ADCS data registry allowed us to apply very strict matching criteria in order to minimize patient-level differences, controlling for far more observed patient-level covariate differences between those undergoing cardiac surgery with TEE vs without TEE. Second, the size of this STS cohort afforded us the opportunity to undertake within-hospital, within-surgeon matches. By creating matched pairs of 2 patients (1 with TEE vs 1 without TEE) admitted to the same hospital, and operated on by the same surgeon, we reduced hospital-level, and surgeon-level, unobserved confounding that could have biased our results. Third, in this study the exposure variable of TEE was found to be a true, intraoperative TEE; an improvement on our previous that could only identify a TEE within a hospitalization.^15,16,32^ Fourth, by performing comprehensive sensitivity analyses, we were able to quantify how much residual, unobserved confounding would be required to alter the conclusions of our analyses. Across all analyses, our findings indicate an association between TEE and improved perioperative outcomes after open cardiac valve or proximal aortic surgery.

Although this matched retrospective observational study cannot elucidate the exact reasons for the clinical outcomes benefit observed with intraoperative TEE, it is likely that intraoperative TEE is conferring some degree of benefit because the association persisted on the strict, within-hospital, within-surgeon matched comparisons. Diagnostic information provided by TEE, interpreted by an experienced echocardiographer—cardiologist or anesthesiologist—could identify surgical complications^5,6,7,8,9^ and improve outcomes by facilitating informed intraoperative decision making by the cardiac surgeon.^5,6,7^ For instance, in valve surgery, paravalvular regurgitation identified by TEE after valve repair or replacement could prompt an immediate valve revision^5,6^ and reduce the risk of reoperation (along with the complications associated with a second surgery). Additionally, TEE imaging can reduce the risk of stroke from air embolism by ensuring the dissipation intracardiac air prior to separation from cardiopulmonary bypass^33^ or decrease the incidence of embolic stroke by ensuring an aortic cannulation or aortic cross clamp site does not embolize atheromatous plaque.^33,34,35,36^ But equal to diagnostic information provided by the TEE imaging itself, it is possible that the association between intraoperative TEE and improved outcomes in this study could be related to the availability of an experienced cardiologist or anesthesiologist certified to perform and interpret a TEE in the operating room.

### Limitations

Our study must be interpreted with awareness of its limitations. First, the observational, nonrandomized design of this study cannot confirm a causal link between TEE and improved clinical outcomes because of the inability to completely eliminate residual confounding; particularly related to inherent differences among those who did not receive TEE. For instance, residual unobserved confounding could be introduced by anatomical considerations at the patient-level or differences in intraoperative management and TEE performance at the clinician-level that might indicate systematic differences among those who did not receive TEE compared with those who did receive TEE. An example of patient-level confounding could be introduced by our inability to exclude patients with anatomical contraindications to TEE such as esophageal (eg, esophagectomy, varices, or strictures)^37^ or gastric (eg, previous gastric bypass surgery, gastric ulcer, or hiatal hernia)^37^ diagnoses. But given the consistent results across all analyses, and the rare prevalence of these diagnoses (<0.5%),^37^ we are reassured that residual patient-level confounding would not change the stated results. An example of clinician-level confounding could be related to the availability of a clinician with the specialization to perform an intraoperative TEE (eg, cardiologist or anesthesiologist). This example of clinician-level confounding could have persisted even after the within-hospital, within-surgeon match because STS data does not identify the clinician performing the intraoperative TEE. Second, while the within-hospital, within-surgeon matched completely controlled for TEE preference by surgical type because we exactly matched on all 9 surgical procedures, there is the possibility that we could not fully adjust for a surgeon who might have variability in TEE preference within the same surgery. For instance, a surgeon who would request TEE for a complex mitral repair, but not request TEE for a more straightforward mitral repair. Third, the 30-day mortality on the within-hospital, within-surgeon matched comparison was 1% to 2% lower than the all-patient, across-hospital, across-surgeon matched comparison. This difference in mortality could be an indication that comparing only surgeons with a probability for intraoperative TEE between 0.30 and 0.70 may represent a different, less sick, patient population compared with the all-patient, across-hospital, across-surgeon match. Fourth, fewer than 23% of patients receiving TEE are included in the matched analyses which potentially limits the generalizability of the stated results. Nevertheless, because results were similar across all analyses—including comprehensive sensitivity analyses—we are reassured of the robustness of the stated results indicating a clinical outcomes benefit to the use of TEE in cardiac valve or aortic surgery.

The current study, particularly in combination with recent observational research demonstrating a consistent outcomes benefit to the use of TEE during cardiac valve^15^ and CABG surgery^14,16^ may have important health policy implications. Because lack of equipoise, it is unlikely that a randomized controlled trial comparing TEE vs no TEE among cardiac surgical patients undergoing cardiac valve or aortic surgery would ever be conducted. Thus, rigorous observational studies such as the current work and previous work^15,16^ are required to inform future AHA/ACC guideline recommendations for the routine use of TEE in cardiac surgery.

## Conclusions

This cohort study found that the use of intraoperative TEE was associated with a lower 30-day mortality and a lower incidence of stroke or 30-day mortality among patients undergoing open cardiac valve or aortic surgery. These findings provide evidence to support the routine use of intraoperative TEE in all open cardiac valve and proximal aortic surgical procedures.

## Notes

Supplement. **eAppendix 1.** Descriptive Statistics of the Study Cohort **eAppendix 2.** Definition of Primary, Secondary, and Negative Control Outcomes **eAppendix 3.** Variability in TEE Preference **eAppendix 4.** Details on Statistical Matching Methodology **eAppendix 5.** Covariate Balance After Matching **eAppendix 6.** Additional Details on Outcome Analysis **eAppendix 7.** Subgroup Analysis **eAppendix 8.** Sensitivity Analysis I: Assessing Robustness of Primary Outcome Findings to Unmeasured Confounding **eAppendix 9.** Sensitivity Analysis II: Assessing Robustness of Results to the Missingness of the TEE Status **eAppendix 10.** Details On the Negative Control Outcome **eAppendix 11.** R and Stata Code
eReferences
