# Supplementary material for: Association of Intraoperative Transesophageal Echocardiography and Clinical Outcomes After Open Cardiac Valve or Proximal Aortic Surgery
Source: JAMA Netw Open. 2022 Feb 9;5(2):e2147820. doi: 10.1001/jamanetworkopen.2021.47820 (PMC8829659; doi:10.1001/jamanetworkopen.2021.47820)
Supplement: Supplement. — eAppendix 1. Descriptive Statistics of the Study Cohort eAppendix 2. Definition of Primary, Secondary, and Negative Control Outcomes eAppendix 3. Variability in TEE Preference eAppendix 4. Details on Statistical Matching Methodology eAppendix 5. Covariate Balance After Matching eAppendix 6. Additional Details on Outcome Analysis eAppendix 7. Subgroup Analysis eAppendix 8. Sensitivity Analysis I: Assessing Robustness of Primary Outcome Findings to Unmeasured Confounding eAppendix 9. Sensitivity Analysis II: Assessing Robustness of Results to the Missingness of the TEE Status eAppendix 10. Details On the Negative Control Outcome eAppendix 11. R and Stata Code eReferences [file jamanetwopen-e2147820-s001.pdf]

Supplemental Online Content

MacKay EJ, Zhang B, Augoustides JG, Groeneveld PW, Desai ND. Association of intraoperative transesophageal echocardiography and clinical outcomes after open cardiac valve or proximal aortic surgery. *JAMA Netw Open.* 2022;5(2):e2147820. doi:10.1001/jamanetworkopen.2021.47820

eAppendix 1. Descriptive Statistics of the Study Cohort

eAppendix 2. Definition of Primary, Secondary, and Negative Control Outcomes

eAppendix 3. Variability in TEE Preference

eAppendix 4. Details on Statistical Matching Methodology

eAppendix 5. Covariate Balance After Matching

eAppendix 6. Additional Details on Outcome Analysis

eAppendix 7. Subgroup Analysis

eAppendix 8. Sensitivity Analysis I: Assessing Robustness of Primary Outcome Findings to Unmeasured Confounding

eAppendix 9. Sensitivity Analysis II: Assessing Robustness of Results to the Missingness of the TEE Status

eAppendix 10. Details On the Negative Control Outcome

eAppendix 11. R and Stata Code

eReferences

This supplemental material has been provided by the authors to give readers additional information about their work.

Contents

1 eAppendix 1. Descriptive Statistics of the Study Cohort 4

1.1 Baseline Characteristics of the Study Cohort 4

1.2 TEE vs. Surgery Type 7

2 eAppendix 2. Definition of Primary, Secondary, and Negative Control Outcomes 8

3 eAppendix 3. Variability in TEE Preference 9

3.1 Hospitals' Preference for TEE 9

3.1.1 Hospitals' Overall Preference for TEE 9

3.1.2 Hospitals' Preference for TEE by Geographic Region 10

3.2 Surgeons' Preference for TEE 11

3.2.1 Surgeons' Overall Preference for TEE 11

3.2.2 Surgeons' Preference for TEE by Region 12

4 eAppendix 4. Details on Statistical Matching Methodology 13

4.1 Glossary of Matching Terms 13

4.2 All-Patient Matched Comparison 14

4.2.1 Study Design 14

4.2.2 Implementation Details 14

4.3 Two Within-Surgeon, Within-Hospital Matches 15

4.3.1 Study Design 15

4.3.2 Implementation Details 16

5 eAppendix 5. Covariate Balance After Matching 17

5.1 All-Patient Matched Comparison 17

5.2 Within-Equivocal-Surgeon, Within-Hospital Matched Comparison 19

5.3 Complementary Within-All-Surgeon, Within-Hospital Matched Comparison 21

6 eAppendix 6. Additional Details on Outcome Analysis 23

6.1 All-Patient Matched Comparison 23

6.1.1 Primary Outcome: 30-Day Mortality 23

6.1.2 Secondary Outcome I: 30-Day Mortality or Incidence of Stroke 23

6.1.3 Secondary Outcome II: 30-Day Mortality or Reoperation 24

6.1.4 Negative Control Outcome: Post-Surgery Creatinine 24

6.2 Within-Equivocal-Surgeon, Within-Hospital Matched Comparison 25

6.2.1 Primary Outcome: 30-Day Mortality 25

6.2.2 Secondary Outcome I: 30-Day Mortality or Incidence of Stroke 25

6.2.3 Secondary Outcome II: 30-Day Mortality or Reoperation 26

6.2.4 Negative Control Outcome: Post-Surgery Creatinine 26

6.3 Within-All-Surgeon, Within-Hospital Matched Comparison 27

6.3.1 Primary Outcome: 30-Day Mortality 27

6.3.2 Secondary Outcome I: 30-Day Mortality or Incidence of Stroke 27

6.3.3 Secondary Outcome II: 30-Day Mortality or Reoperation 28

6.3.4 Negative Control Outcome: Post-Surgery Creatinine 28

6.4 Summary ... 28

7 eAppendix 7. Subgroup analysis **30**

7.1 Defining subgroups ... 30

8 eAppendix 8. Sensitivity Analysis I: Assessing Robustness of Primary Outcome Findings to Un- measured Confounding **33**

9 eAppendix 9. Sensitivity Analysis II: Assessing Robustness of Results to the Missingness of the TEE Status **34**

10 eAppendix 10. Details On the Negative Control Outcome **35**

10.1 Rationale Behind Negative Control Outcome Selection ... 35

10.2 Comment on Results of Negative Control ... 35

11 eAppendix 11. R and Stata Code **37**

1 eAppendix 1. Descriptive Statistics of the Study Cohort

1.1 Baseline Characteristics of the Study Cohort

After applying the inclusion/exclusion criteria, we are left with n = 872, 936 patients. eTable 1 (Part I), 2 (Part II), and 3 (Part III) summarize baseline covariates of the study cohort, including demographics, preexisting comorbid conditions, laboratory values, surgical variables, surgery type, and predicted risk scores.

eTable 1: Balance table before matching: Part I. Mean (SD) are reported for continuous variables and number of counts (%) for binary, categorical, and ordinal variables.

	All Patients (n = 872,936)	Patients with TEE (n = 711,326)	Patients without TEE (n = 161,610)
**Demographics**			
Age	65.61 (13.17)	65.49 (13.17)	66.14 (13.17)
BSA	1.97 (0.26)	1.97 (0.26)	1.96 (0.26)
BSA missing (%)	1539 (0.18)	1135 (0.16)	404 (0.25)
Height (cm)	170.87 (11.08)	170.93 (11.05)	170.64 (11.21)
Height missing (%)	1461 (0.17)	1079 (0.15)	382 (0.24)
Weight (kg)	85.35 (20.80)	85.34 (20.78)	85.39 (20.88)
Weight missing (%)	1359 (0.16)	1013 (0.14)	346 (0.21)
BMI	29.32 (10.56)	29.29 (10.44)	29.47 (11.04)
BMI missing (%)	1539 (0.18)	1135 (0.16)	404 (0.25)
Male (%)	540229 (61.89)	440033 (61.86)	100196 (62.00)
White (%)	742384 (85.04)	603895 (84.90)	138489 (85.69)
Black (%)	63565 (7.28)	52062 (7.32)	11503 (7.12)
**Admission**			
ER (%)	111673 (12.79)	89325 (12.56)	22348 (13.83)
Transfer (%)	109005 (12.49)	89739 (12.62)	19266 (11.92)
Elective & Others (%)	652258 (74.72)	532262 (74.83)	119996 (74.25)
**Preexisting Comorbidities**			
Diabetes (%)	245620 (28.14)	198959 (27.97)	46661 (28.87)
Dyslip (%)	601086 (68.86)	491110 (69.04)	109976 (68.05)
Dialysis (%)	25377 (2.91)	20672 (2.91)	4705 (2.91)
NYH Class (%)			
0	508307 (58.23)	409428 (57.56)	98879 (61.18)
1	21753 (2.49)	18357 (2.58)	3396 (2.10)
2	107990 (12.37)	89665 (12.61)	18325 (11.34)
3	161027 (18.45)	132911 (18.68)	28116 (17.40)
4	73859 (8.46)	60965 (8.57)	12894 (7.98)
Hypertension (%)	678783 (77.76)	553131 (77.76)	125652 (77.75)
InfEndo (%)	71824 (8.23)	59482 (8.36)	12342 (7.64)
HmO2 (%)	23604 (2.70)	19763 (2.78)	3841 (2.38)
SlpApn (%)	125048 (14.32)	105016 (14.76)	20032 (12.40)
Liver diseases (%)	39977 (4.58)	33762 (4.75)	6215 (3.85)
Cancer (%)	45663 (5.23)	37811 (5.32)	7852 (4.86)
PVD (%)	93417 (10.70)	75958 (10.68)	17459 (10.80)
CVD (%)	149725 (17.15)	123290 (17.33)	26435 (16.36)
CVA (%)	74233 (8.50)	60958 (8.57)	13275 (8.21)

eTable 2: Balance table before matching: Part II. Mean (SD) are reported for continuous variables and number of counts (%) for binary, categorical, and ordinal variables.

	All Patients (n = 872,936)	Patients with TEE (n = 711,326)	Patients without TEE (n = 161,610)
**Hemodynamic Data & Laboratory Values**			
PASYS	40.86 (11.59)	40.89 (11.81)	40.74 (10.57)
PASYS missing (%)	390866 (44.78)	303856 (42.72)	87010 (53.84)
Ejection fraction	55.23 (11.91)	55.24 (11.90)	55.16 (11.95)
Ejection fraction missing (%)	30789 (3.53)	21533 (3.03)	9256 (5.73)
Creatinine	1.18 (1.02)	1.18 (1.02)	1.19 (1.00)
Creatinine missing (%)	4024 (0.46)	2657 (0.37)	1367 (0.85)
Albumin	3.78 (0.56)	3.79 (0.57)	3.77 (0.56)
Albumin missing (%)	149541 (17.13)	112273 (15.78)	37268 (23.06)
MELD score	9.26 (3.39)	9.25 (3.40)	9.30 (3.30)
MELD score missing (%)	193713 (22.19)	148190 (20.83)	45523 (28.17)
**Surgical Variables**			
Previous CABG surgery (%)	45886 (5.26)	36607 (5.15)	9279 (5.74)
Previous valve surgery (%)	81237 (9.31)	66877 (9.40)	14360 (8.89)
IABP placed (%)	39168 (4.49)	31077 (4.37)	8091 (5.01)
CathtoSurg	34.61 (55.18)	35.32 (55.68)	31.49 (52.82)
CathtoSurg missing (%)	110502 (12.66)	85114 (11.97)	25388 (15.71)
CPB	138.81 (64.82)	138.45 (64.03)	140.38 (68.15)
CPB missing (%)	3580 (0.41)	2129 (0.30)	1451 (0.90)
Time in OR	356.59 (118.80)	356.40 (117.72)	357.43 (123.44)
Time in OR missing (%)	3034 (0.35)	2453 (0.34)	581 (0.36)
Time of skin incicion	267.53 (106.19)	267.00 (105.26)	269.86 (110.14)
Time of skin incicion missing (%)	5067 (0.58)	3297 (0.46)	1770 (1.10)
Single valve (%)	808010 (92.56)	656941 (92.35)	151069 (93.48)
**Surgery Type**			
AV repair (%)	13846 (1.59)	11625 (1.63)	2221 (1.37)
AV replacement (%)	514712 (58.96)	412751 (58.03)	101961 (63.09)
MV repair (%)	179969 (20.62)	154423 (21.71)	25546 (15.81)
MV replacement (%)	155182 (17.78)	126991 (17.85)	28191 (17.44)
Tricuspid valve repair/replacement (%)	79420 (9.10)	67817 (9.53)	11603 (7.18)
Pulmonic valve repair/replacement (%)	4790 (0.55)	3914 (0.55)	876 (0.54)
Aortic root/valved conduit (Bentall) (%)	37658 (4.31)	30715 (4.32)	6943 (4.30)
Aortic valve sparing root (%)	35569 (4.07)	29946 (4.21)	5623 (3.48)
Aortic homograft or non-valved conduit (%)	13784 (1.58)	10926 (1.54)	2858 (1.77)
Plus CABG (%)	270991 (31.04)	217360 (30.56)	53631 (33.19)
Plus other cardiac surgery (%)	245173 (28.09)	204702 (28.78)	40471 (25.04)
**Valve Surgery Volume**			
Hospital-level	2365.80 (3401.06)	2442.03 (3497.25)	2030.32 (2916.90)
Surgeon-level	648.79 (552.56)	667.31 (566.87)	567.29 (476.10)

eTable 3: Balance table before matching: Part III. Mean (SD) are reported for continuous variables and number of counts (%) for binary, categorical, and ordinal variables.

	All Patients (n = 872,936)	Patients (TEE) (n = 711,326)	Patients (no TEE) (n = 161,610)
**Predicted Risk Scores (4 digits)**			
PredMort	0.0365 (0.0396)	0.0363 (0.0392)	0.0377 (0.0417)
PredMort missing (%)	299703 (34.33)	247381 (34.78)	52322 (32.38)
PredDeep	0.0034 (0.0031)	0.0034 (0.0031)	0.0036 (0.0030)
PredDeep missing (%)	299701 (34.33)	247380 (34.78)	52321 (32.37)
PredReop	0.0782 (0.0361)	0.0775 (0.0359)	0.0815 (0.0368)
PredReop missing (%)	299699 (34.33)	247378 (34.78)	52321 (32.37)
PredStro	0.0179 (0.0105)	0.0178 (0.0105)	0.0182 (0.0107)
PredStro missing (%)	299699 (34.33)	247378 (34.78)	52321 (32.37)
PredVent	0.1428 (0.1061)	0.1421 (0.1055)	0.1461 (0.1084)
PredVent missing (%)	299699 (34.33)	247378 (34.78)	52321 (32.37)
PredRenF	0.0523 (0.0486)	0.0518 (0.0482)	0.0545 (0.0503)
PredRenF missing (%)	315517 (36.14)	260120 (36.57)	55397 (34.28)
PredMM	0.2069 (0.1174)	0.2058 (0.1169)	0.2120 (0.1196)
PredMM missing (%)	299699 (34.33)	247378 (34.78)	52321 (32.37)
Pred6D	0.3311 (0.1526)	0.3326 (0.1530)	0.3247 (0.1503)
Pred6D missing (%)	299700 (34.33)	247379 (34.78)	52321 (32.37)
Pred14D	0.1051 (0.0828)	0.1047 (0.0827)	0.1070 (0.0834)
Pred14D missing (%)	299700 (34.33)	247379 (34.78)	52321 (32.37)

1.2 TEE vs. Surgery Type

eTable 4: TEE proportion by surgery type

**Surgery Type**	Number of Patients	Percentage with TEE	Percentage without TEE
AV repair	13846	84.0%	16.0%
AV replacement	514712	80.2%	19.8%
MV repair	179969	85.8%	14.2%
MV replacement	155182	81.8%	18.2%
Tricuspid valve repair/replacement	79420	85.4%	14.6%
Pulmonic valve repair/replacement	4790	81.7%	18.3%
Aortic root/valved conduit (Bentall)	37658	81.6%	18.4%
Aortic valve sparing root	35569	84.2%	15.8%
Aortic homograft or non-valved conduit	13784	79.3%	20.7%

2 eAppendix 2. Definition of Primary, Secondary, and Negative Control Outcomes

All outcome variable labels were consistent across all three Society of Thoracic Surgeons (STS), Adult Cardiac Surgery Database (ACSD) versions (2.73, 2.81, and 2.90).

1. Primary Outcome: 30-day mortality
   (a) 30-day mortality
      i. Long name: "Mort-30d Status"
      ii. Short name: "Mt30Stat"
2. Secondary Outcome: Stroke (or 30-day mortality)
   (a) Stroke
      i. Long name: "Post-Op-Neuro-Stroke Perm"
      ii. Short name: "CNStrokP"
3. Secondary Outcome: Reoperation composite: return to operating room for: bleed, valve reintervention, or graft bypass reintervention; (or 30-day mortality)
   (a) Return to operating room for bleeding
      i. Long name: "Post-Op-ReOp Bleed"
      ii. Short name: "COpReBld"
   (b) Return to operating room for valve reintervention
      i. Long name: "Post-Op-Reop Vlv Dys"
      ii. Short name: "COpReVlv"
   (c) Return to operating room for coronary graft reintervention
      i. Long name: "Post-Op-Reintervention-Graft Occlusion"
      ii. Short name: "COpReGft"
4. Negative Control Outcome: Postoperative Change in Creatinine (defined as the difference between postoperative creatinine and preoperative creatinine)
   (a) Postoperative Creatinine
      i. Long name: "Postoperative Creatinine Level"
      ii. Short name: "PostCreat"
   (b) Preoperative Creatinine Level
      i. Long name: "RF-Last Creat Level"
      ii. Short name: "CreatLst"

3 eAppendix 3. Variability in TEE Preference

3.1 Hospitals' Preference for TEE

We explore hospitals' preference for using TEE during valve surgeries in this section. eFigure 1 plots the overall distribution of hospitals' preference (TEE fraction), and eFigure 2 presents boxplots of hospitals' preference by geographic region.

3.1.1 Hospitals' Overall Preference for TEE

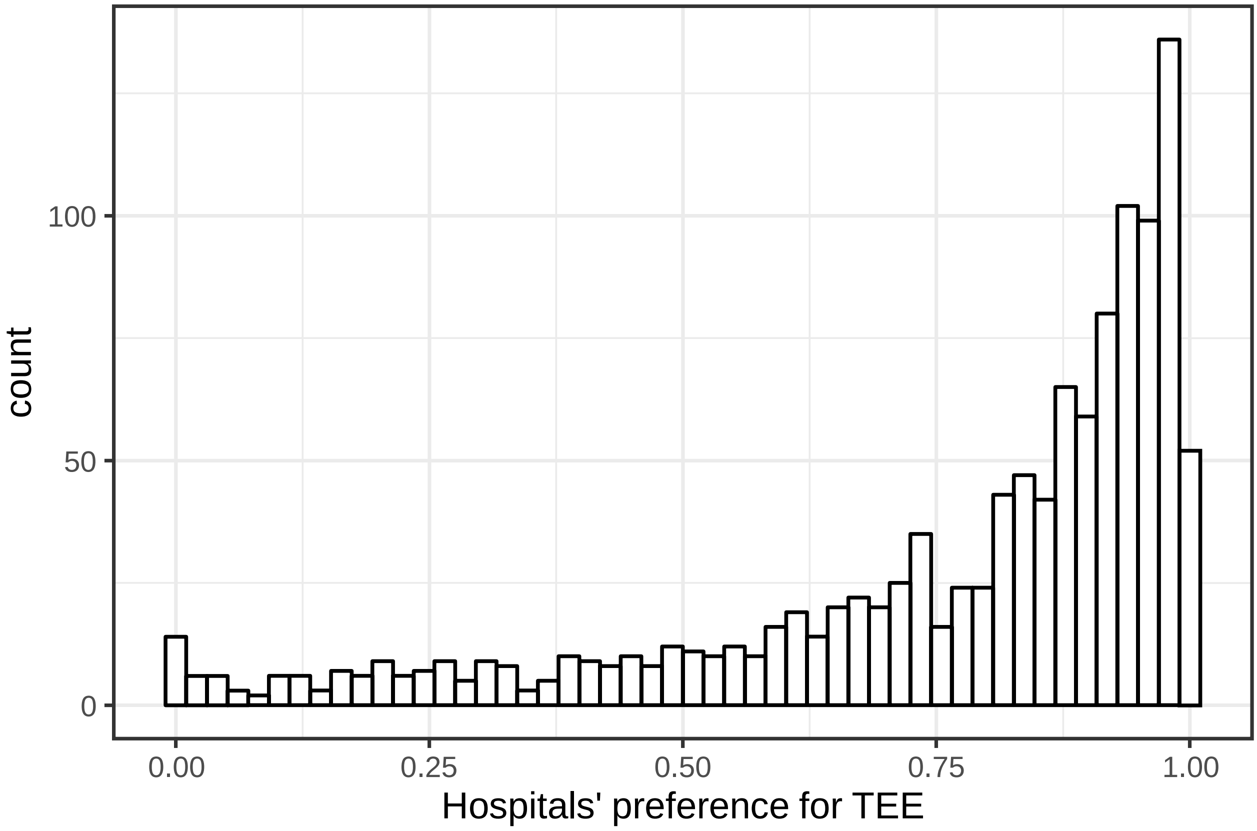

eFigure 1: Hospitals' preference for TEE

3.1.2 Hospitals' Preference for TEE by Geographic Region

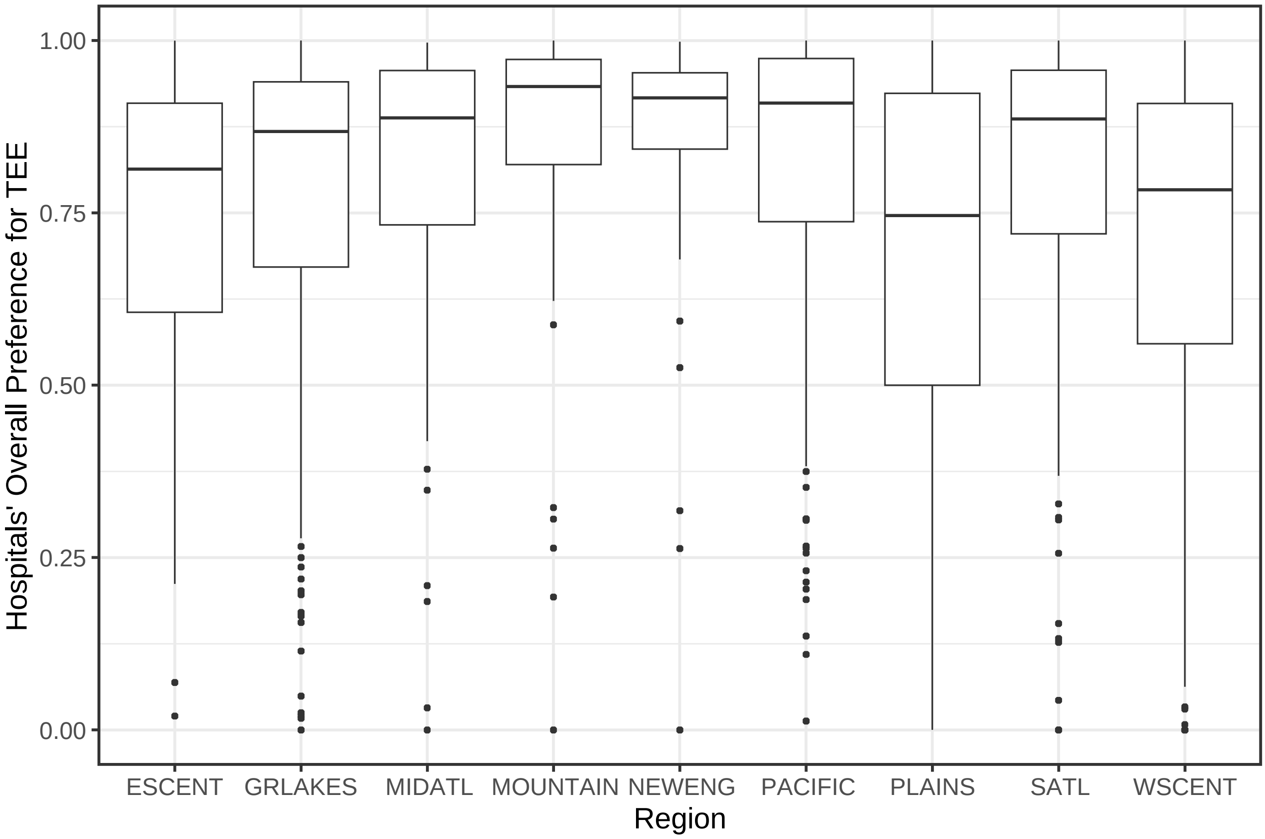

eFigure 2: Hospitals' overall preference for TEE by geographic region

3.2 Surgeons' Preference for TEE

We explore surgeons' preference for using TEE during valve surgeries in this section. eFigure 3 plots the overall distribution of surgeons' preference (TEE fraction), and eFigure 4 presents the boxplots of surgeons' preference by geographic region.

3.2.1 Surgeons' Overall Preference for TEE

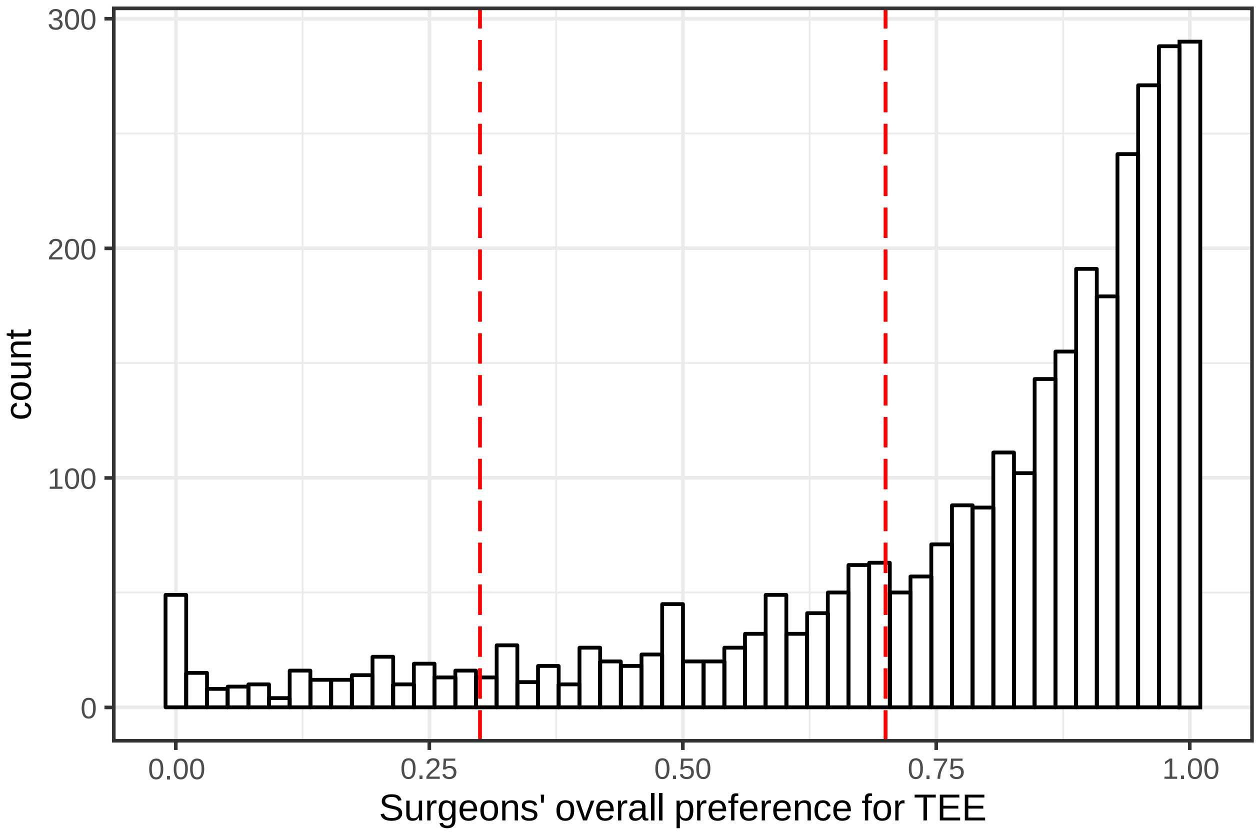

eFigure 3: Surgeons' preference for TEE. Out of 3159 surgeons, 595 of them have preference between 0.30 and 0.70.

3.2.2 Surgeons' Preference for TEE by Region

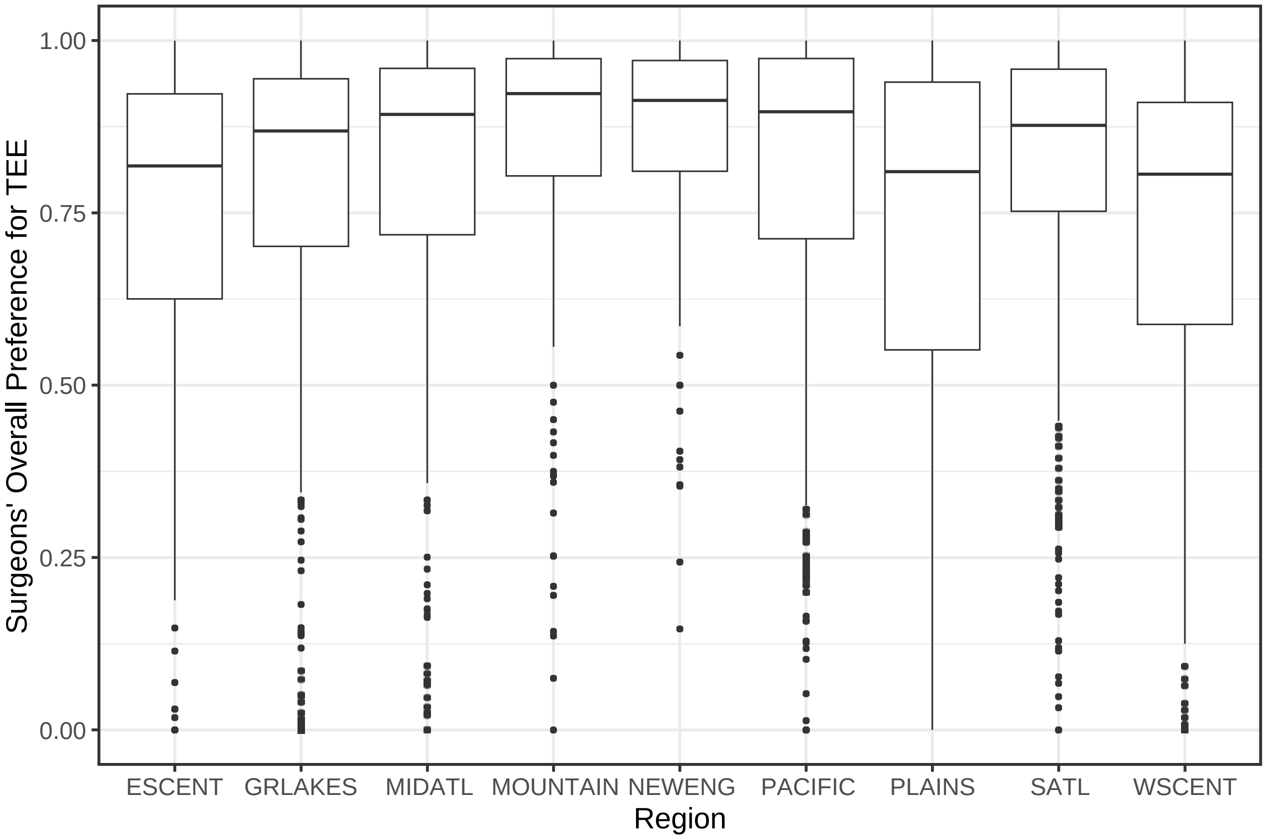

eFigure 4: Surgeons' overall preference for TEE by region

4 eAppendix 4. Details on Statistical Matching Methodology

Statistical matching is a commonly used method to adjust for observed covariates and embed observational data into an approximately randomized experiment (Rosenbaum, 2002, 2010). We define terminologies in Section 4.1, give details of the all-patient matched comparison in Section 4.2, and details of two within-surgeon, within-hospital matches in Section 4.3.

4.1 Glossary of Matching Terms

Bipartite Matching: Matching cases to controls based on a binary treatment status.

Optimal (Bipartite) Matching: Match cases to controls in an optimal way such that some properly defined total cases-to-control distances is minimized after matching.

Propensity Score: The propensity score is the conditional probability of assignment to a particular treatment given a vector of observed covariates (Rosenbaum and Rubin, 1983).

Optimal Matching Within Propensity Score Calipers: A hybrid matching method that minimizes the total cases-to-control distances subject to the constraint that matched cases and control units differ in their estimated propensity scores by no more than a value known as the "caliper" (Rosenbaum and Rubin, 1985). Rosenbaum and Rubin (1985) found that this hybrid method is superior to metric-based matching and propensity score matching.

Mahalanobis Distance: A multivariate measure of covariate distance between units in a sample (Mahalanobis, 1936, Rubin, 1980). The squared Mahalanobis distance is equal to the difference in covariate values of treated units and matched control units, divided by the covariate's standard deviation. Mahalanobis distance takes into account the correlation structure among covariates. The distance is zero if two units have the same value for all covariates and increases as two units become more dissimilar.

Exact Matching: Matching cases to controls requiring the same value of a nominal covariate (Rosenbaum, 2002).

Fine Balance: A matching technique that balances exactly the marginal distribution of one nominal variable or the joint distribution of several nominal variables in the treated and control groups after matching (Rosenbaum et al., 2007; Yu et al., 2020).

4.2 All-Patient Matched Comparison

4.2.1 Study Design

In the all-patient matched comparison, each of the 161, 610 patients undergoing valve surgery without TEE in the study cohort is matched to one patient undergoing valve surgery with TEE. We matched exactly on

1. New York Heart classification: 0 (none), 1, 2, 3, 4;
2. Predicted mortality rate quartiles and missingness: NA, 1st, 2nd, 3rd, 4th,

finely balanced the joint distribution of the following nominal variables (Rosenbaum et al., 2007):

1. An indicator for AV replacement (1/0);
2. An indicator for MV replacement (1/0);
3. An indicator for aortic proximal (1/0);
4. An indicator for CABG (1/0);
5. An indicator for other cardiac surgery (1/0);
6. An indicator for race/ethnicity being white (1/0);
7. An indicator for emergency room admission (1/0);
8. An indicator for trasfer admission (1/0);
9. An indicator for normal ejection fraction (1/0);
10. An indicator for preexisting hypertension (1/0);
11. An indicator for having a valve surgery before (1/0),

and balanced all other variables under the demographics, admission, preexisting comorbidities, hemodynamic data & laboratory values, surgical variables, and surgery type categories in eTable 1 and 2. We further matched on surgery-level and hospital-level valve surgery volume.

4.2.2 Implementation Details

Within each of the $5 \times 5 = 25$ strata formed by New York Heart classification and predicted mortality rate quartiles, we used a version of the state-of-the-art "optimal matching within propensity score calipers" algorithm (Rosenbaum and Rubin, 1985; Rosenbaum, 1989) designed for large administrative databases implemented in the R package bigmatch (Yu et al., 2020). Below, we discuss three implementation details:

1. The propensity score was estimated via a standard ridge-type logistic regression using the state-of-the-art R package glmnet (Friedman et al., 2010) on all study samples.

2. Under the "optimal matching within a propensity score caliper" regime, the size of caliper is important because too large a caliper size renders the matching problem computationally challenging, while too small a caliper size renders the matching problem infeasible (i.e., we cannot match each no-TEE unit with one TEE unit). Fortunately, function optcal in the package bigmatch is able to determine the smallest caliper size such that the matching problem remains feasible using a so-called Glover's algorithm (Yu et al., 2020). We therefore used a caliper size equal to $E + 0.05$ where E is the smallest caliper size such that the matched problem remains feasible as determined by the function optcal in each of the 25 strata, and we added 0.05 to allow some flexibility.

3. Function nfmatch allows researchers to pursue "optimal propensity score matching" while finely balancing the joint distribution of a list of pre-specified categorical variables at the same time. To do this, we set option fine in the function nfmatch to the list of 11 variables to be finely balanced specified in the Section 4.2.1.

See Section 11 for R code implementing the match.

4.3 Two Within-Surgeon, Within-Hospital Matches

4.3.1 Study Design

In a second study design, we consider matching patients of the *same* surgeon. Since the same surgeon may practice in different hospitals, we further match exactly on the hospital ID. In other words, each matched pair consists of two patients of *the same surgeon in the same hospital*, one undergoing valve surgery with TEE and the other not. This design may maximally control for the selection bias due to surgeon and/or hospital-level residual confounding.

We considered two variants of this design. Our primary within-surgeon, within-hospital match only considered patients whose surgeons had an overall preference between 30% and 70% for using TEE during valve surgeries. Moreover, in addition to matching exactly on the surgeon ID and hospital ID, we further match exactly on the surgery type (including having multiple surgery types, i.e., a TEE patient with an AV replacement surgery plus a CABG surgery was matched to a no-TEE patient with an AV replacement surgery and plus a CABG surgery), ejection fraction being normal (55% to 70%), New York Heart classification, and quartiles of predicted mortality. We summarize below variables being *exactly* matched upon:

1. Surgeon ID;

2. Hospital ID;

3. An indicator of ejection fraction being normal (1/0);

4. New York Heart classification: 0 (none), 1, 2, 3, 4;

5. Predicted mortality rate quartiles and missingness: NA, 1st, 2nd, 3rd, 4th;

6. An indicator for AV repair (1/0);

7. An indicator for AV replacement (1/0);

8. An indicator for MV repair (1/0);

9. An indicator for MV replacement (1/0);
10. An indicator for tricuspid valve repair/replacement (1/0);
11. An indiator for pulmonic valve repair/replacement (1/0);
12. An indicator for aortic proximal (Aortic root/valved conduit (Bentall), AV sparing root, or Aortic homograft or non-valved conduit) (1/0);
13. An indicator for plus CABG (1/0);
14. An indicator for plus other cardiac surgery (1/0).

We also balanced all other variables under the demographics, admission, preexisting comorbidities, hemodynamic data & laboratory values, surgical variables, and surgery type categories in eTable 1 and 2.

As a complementary analysis, we further considered a within-surgeon, within-hospital match that utilized all surgeons.

4.3.2 Implementation Details

Within each stratum formed by surgeon ID and hospital ID, we used a statistical matching algorithm called optimal subset matching (Rosenbaum, 2012, 2020) implemented in the `R` package `rcbsubset`. We implemented the matching algorithm with all tuning parameters set to their *default* values.

See Section 11 for `R` code implementing the match.

5 eAppendix 5. Covariate Balance After Matching

5.1 All-Patient Matched Comparison

eTable 5: Balance Table After Matching: Part I

	TEE = 0 (n = 161,610)	TEE = 1 (n = 161,610)	Std. Mean Difference
**Demographics**			
Age	66.14 (13.17)	66.32 (13.01)	0.014
BSA	1.96 (0.26)	1.97 (0.25)	0.022
BSA missing	404 (0.25)	161 (0.10)	-0.036
BMI	29.47 (11.04)	29.16 (8.19)	-0.030
BMI missing	404 (0.25)	161 (0.10)	-0.036
Male (%)	100196 (62.00)	103109 (63.80)	0.037
White (%)	138489 (85.69)	138678 (85.81)	0.003
Black (%)	11503 (7.12)	11427 (7.07)	-0.002
**Admission Type** (%)			
ER	22348 (13.83)	22274 (13.78)	-0.001
Transfer	19266 (11.92)	19309 (11.95)	0.001
Elective & Others	119996 (74.25)	120027 (74.27)	0.000
**Preexisting Comorbidities**			
Diabetes (%)	46661 (28.87)	43903 (27.17)	-0.038
Dyslip (%)	109976 (68.05)	114370 (70.77)	0.059
Dialysis (%)	4705 (2.91)	4163 (2.58)	-0.020
Hypertension (%).	125652 (77.75)	125745 (77.81)	0.001
NYH Class (%)			
None	98879 (61.18)	98879 (61.18)	0
1	3396 (2.10)	3396 (2.10)	0
2	18325 (11.34)	18325 (11.34)	0
3	28116 (17.40)	28116 (17.40)	0
4	12894 (7.98)	12894 (7.98)	0
InfEndo (%)	12342 (7.64)	12092 (7.48)	-0.006
HmO2 (%)	3841 (2.38)	2765 (1.71)	-0.041
SlpApn (%)	20032 (12.40)	21413 (13.25)	0.024
Liver diseases (%)	6215 (3.85)	5056 (3.13)	-0.034
Cancer (%)	7852 (4.86)	6646 (4.11)	-0.034
PVD (%)	17459 (10.80)	17678 (10.94)	0.004
CVD (%)	26435 (16.36)	27383 (16.94)	0.015
CVA (%)	13275 (8.21)	14799 (9.16)	0.034

eTable 6: Balance Table After Matching: Part II.

	TEE = 0 (n = 161,610)	TEE = 1 (n = 161,610)	Std. Mean Difference
**Hemodynamic Data & Laboratory Values**			
PASYS	40.74 (10.57)	40.61 (10.11)	-0.011
PASYS missing (%)	87010 (53.84)	87539 (54.17)	0.007
Ejection fraction	55.16 (11.95)	54.95 (11.56)	-0.018
EF missing (%)	9256 (5.73)	7782 (4.82)	-0.050
EF normal (%)	98157 (60.74)	98242 (60.79)	0.001
Creatinine	1.19 (1.00)	1.18 (0.98)	-0.011
Creatinine missing (%)	1367 (0.85)	667 (0.41)	-0.064
Albumin	3.77 (0.56)	3.77 (0.53)	0.007
Albumin missing (%)	37268 (23.06)	34616 (21.42)	-0.044
MELD score	9.30 (3.30)	9.17 (3.20)	-0.037
MELD score missing (%)	45523 (28.17)	41738 (25.83)	-0.056
**Surgical Procedures**			
Previous CABG surgery (%)	9279 (5.74)	6887 (4.26)	-0.066
Previous valve surgery (%)	14360 (8.89)	14126 (8.74)	-0.005
IABP placed (%)	8091 (5.01)	5483 (3.39)	-0.078
CathtoSurg	31.49 (52.82)	29.05 (43.77)	-0.044
CathtoSurg missing (%)	25388 (15.71)	21531 (13.32)	-0.072
CBP	140.38 (68.15)	139.81 (63.14)	-0.009
CBP missing (%)	1451 (0.90)	596 (0.37)	-0.083
Time in OR (min)	357.43 (123.44)	353.28 (114.23)	-0.035
Time in OR missing (%)	581 (0.36)	171 (0.11)	-0.043
Time of skin incicion (min)	269.86 (110.14)	267.56 (104.50)	-0.022
Time of skin incicion missing (%)	1770 (1.10)	686 (0.42)	-0.088
Single valve (%)	151069 (93.48)	151667 (93.85)	0.014
**Surgery Type**			
AV repair (%)	2221 (1.37)	2265 (1.40)	0.002
AV replacement (%)	101961 (63.09)	101952 (63.09)	-0.000
MV repair (%)	25546 (15.81)	25548 (15.81)	-0.000
MV replacement (%)	28191 (17.44)	28175 (17.43)	-0.000
Tricuspid valve repair/replacement (%)	11603 (7.18)	9881 (6.11)	-0.037
Pulmonic valve repair/replacement (%)	876 (0.54)	563 (0.35)	-0.026
Aortic root/valved conduit (Bentall) (%)	6943 (4.30)	7182 (4.44)	0.008
Aortic valve sparing root (%)	5623 (3.48)	5406 (3.35)	-0.007
Aortic homograft or non-valved conduit (%)	2858 (1.77)	2452 (1.52)	-0.020
Plus CABG (%)	53631 (33.19)	53500 (33.10)	-0.002
Plus other cardiac surgery (%)	40471 (25.04)	40453 (25.03)	-0.000
**Predicted Mortality**			
Predicted Mortality Quartiles (%)			
NA	52411 (32.43)	52411 (32.43)	0
1	15626 (9.67)	15626 (9.67)	0
2	23780 (14.71)	23780 (14.71)	0
3	33131 (20.50)	33131 (20.50)	0
4	36662 (22.69)	36662 (22.69)	0
Predicted Mortality, %	3.77 (4.17)	3.73 (3.82)	-0.004
**Valve Surgery Volume**			
Surgeon-level	567.29 (476.10)	546.58 (439.14)	-0.037
Hospital-level	2030.32 (2916.90)	1925.52 (2628.68)	-0.031

5.2 Within-Equivocal-Surgeon, Within-Hospital Matched Comparison

eTable 7: Balance Table After Matching: Part I

	TEE = 0 (n = 22,739)	TEE = 1 (n = 22,739)	Std.Diff
**Hospital**			
ID 104367	4	4	0
ID 105306	82	82	0
.	.	.	.
**Demographics**			
Age	66.94 (12.72)	66.89 (12.46)	-0.003
BSA	1.97 (0.25)	1.97 (0.25)	0.013
BSA missing	19 (0.08)	8 (0.04)	-0.012
BMI	29.64 (9.98)	29.57 (8.50)	-0.006
BMI missing	19 (0.08)	8 (0.04)	-0.012
Male (%)	14113 (62.07)	14131 (62.14)	0.002
White (%)	20111 (88.44)	20224 (88.94)	0.014
Black (%)	1287 (5.66)	1239 (5.45)	-0.008
**Admission Type (%)**			
ER	2247 (9.88)	2158 (9.49)	-0.012
Transfer	2172 (9.55)	2300 (10.11)	0.017
Elective & Others	18320 (80.57)	18281 (80.39)	0.004
**Preexisting Comorbidities**			
Diabetes (%)	6650 (29.24)	6687 (29.41)	0.004
Dyslip (%)	16182 (71.16)	16575 (72.89)	0.037
Dialysis (%)	474 (2.08)	477 (2.10)	0.001
Hypertension (%)	17871 (78.59)	18140 (79.77)	0.028
NYH Class (%)			
None	16208 (71.28)	16208 (71.28)	0
1	158 (0.69)	158 (0.69)	0
2	1926 (8.47)	1926 (8.47)	0
3	3389 (14.90)	3389 (14.90)	0
4	1058 (4.65)	1058 (4.65)	0
InfEndo (%)	1208 (5.31)	1243 (5.47)	0.006
HmO2 (%)	405 (1.78)	411 (1.81)	0.002
SlpApn (%)	2601 (11.44)	3258 (14.33)	0.083
Liver diseases (%)	638 (2.81)	704 (3.10)	0.014
Cancer (%)	921 (4.05)	1016 (4.47)	0.019
PVD (%)	2257 (9.93)	2306 (10.14)	0.007
CVD (%)	3290 (14.47)	3519 (15.48)	0.027
CVA (%)	1589 (6.99)	1604 (7.05)	0.002

eTable 8: Balance Table After Matching: Part II

	TEE = 0 (n = 22,739)	TEE = 1 (n = 22,739)	Std.Diff
**Hemodynamic Data & Laboratory Values**			
PASYS	40.38 (10.32)	40.24 (10.72)	-0.012
PASYS missing (%)	12314 (54.15)	11226 (49.37)	-0.097
Ejection fraction	56.21 (11.41)	56.15 (11.36)	-0.005
EF missing (%)	1057 (4.65)	851 (3.74)	-0.049
EF normal (%)	15153 (66.64)	15153 (66.64)	0
Creatinine	1.12 (0.85)	1.13 (0.86)	0.008
Creatinine missing (%)	103 (0.45)	45 (0.20)	-0.038
Albumin	3.82 (0.52)	3.83 (0.52)	0.008
Albumin missing (%)	5763 (25.34)	5038 (22.16)	-0.085
MELD score	9.08 (2.94)	9.07 (2.99)	-0.003
MELD score missing	6379 (28.05)	5616 (24.70)	-0.081
**Surgical Procedures**			
Previous CABG surgery (%)	1254 (5.51)	1121 (4.93)	-0.026
Previous valve surgery (%)	1635 (7.19)	1776 (7.81)	0.021
IABP placed	791 (3.48)	790 (3.47)	-0.000
CathtoSurg	31.83 (51.72)	33.25 (50.82)	0.026
CathtoSurg missing	3031 (13.33)	2330 (10.25)	-0.093
CBP	126.87 (58.96)	125.42 (57.13)	-0.022
CBP missing	200 (0.88)	33 (0.15)	-0.115
Time in OR (min)	331.35 (108.01)	331.54 (106.45)	0.002
Time in OR missing (%)	36 (0.16)	26 (0.11)	-0.007
Time of skin incicion (min)	248.21 (96.15)	247.80 (95.24)	-0.004
Time of skin incicion missing (%)	188 (0.83)	45 (0.20)	-0.083
Single valve (%)	21836 (96.03)	21836 (96.03)	0
**Surgery Type**			
AV repair (%)	163 (0.72)	163 (0.72)	0
AV replacement (%)	16191 (71.20)	16191 (71.20)	0
MV repair (%)	2963 (13.03)	2963 (13.03)	0
MV replacement (%)	2956 (13.00)	2956 (13.00)	0
Tricuspid valve repair/replacement (%)	913 (4.02)	913 (4.02)	0
Pulmonic valve repair/replacement (%)	63 (0.28)	63 (0.28)	0
Aortic root/valved conduit (Bentall) (%)	629 (2.77)	640 (2.81)	0.002
Aortic valve sparing root (%)	449 (1.97)	469 (2.06)	0.004
Aortic homograft or non-valved conduit (%)	261 (1.15)	242 (1.06)	-0.007
Plus CABG (%)	7224 (31.77)	7224 (31.77)	0
Plus other cardiac surgery (%)	4321 (19.00)	4321 (19.00)	0
**Predicted Mortality**			
Predicted Mortality Quartiles (%)			
None	5462 (24.02)	5462 (24.02)	0
1	2714 (11.94)	2714 (11.94)	0
2	4027 (17.71)	4027 (17.71)	0
3	5349 (23.52)	5349 (23.52)	0
4	5187 (22.81)	5187 (22.81)	0
Predicted Mortality, %	3.47 (3.71)	3.45 (3.70)	-0.002
**Valve Surgery Volume**			
Hospital-level	2034.70 (1954.26)	2034.70 (1954.26)	0
Surgeon-level	632.52 (455.16)	632.52 (455.16)	0

5.3 Complementary Within-All-Surgeon, Within-Hospital Matched Comparison

eTable 9: Balance Table After Matching: Part I

	TEE = 0 (n = 65,340)	TEE = 1 (n = 65,340)	Std. Mean Difference
**Hospital**			
ID 100427	24	24	0
ID 102960	4	4	0
ID 103517	12	12	0
:	:	:	.
**Demographics**			
Age	66.65 (12.96)	66.87 (12.67)	0.016
BSA	1.97 (0.25)	1.97 (0.25)	0.014
BSA missing	189 (0.29)	76 (0.12)	-0.041
BMI	29.48 (10.29)	29.37 (9.19)	-0.010
BMI missing	189 (0.29)	76 (0.12)	-0.041
Male (%)	40893 (62.58)	41004 (62.75)	0.003
White (%)	57003 (87.24)	58127 (88.96)	0.048
Black (%)	3943 (6.03)	3317 (5.08)	-0.037
**Admission Type (%)**			
ER	6990 (10.70)	6140 (9.40)	-0.039
Transfer	7364 (11.27)	6835 (10.46)	-0.024
Elective & Others	50986 (78.03)	52365 (80.14)	0.049
**Preexisting Comorbidities**			
Diabetes (%)	18609 (28.48)	18418 (28.19)	-0.007
Dyslip (%)	45477 (69.60)	47051 (72.01)	0.052
Dialysis (%)	1527 (2.34)	1245 (1.91)	-0.026
Hypertension (%)	51159 (78.30)	52311 (80.06)	0.042
NYH Class (%)			
None	46051 (70.48)	46051 (70.48)	0
1	581 (0.89)	581 (0.89)	0
2	5839 (8.94)	5839 (8.94)	0
3	9562 (14.63)	9562 (14.63)	0
4	3307 (5.06)	3307 (5.06)	0
InfEndo (%)	3980 (6.09)	3732 (5.71)	-0.014
HmO2 (%)	1400 (2.14)	1219 (1.87)	-0.017
SlpApn (%)	8267 (12.65)	9462 (14.48)	0.052
Liver diseases (%)	2307 (3.53)	2079 (3.18)	-0.017
Cancer (%)	3190 (4.88)	2961 (4.53)	-0.016
PVD (%)	7226 (11.06)	7114 (10.89)	-0.001
CVD (%)	10603 (16.23)	10864 (16.63)	0.011
CVA (%)	5163 (7.90)	5153 (7.89)	-0.001

eTable 10: Balance Table After Matching: Part II

	TEE = 0 (n = 65,340)	TEE = 1 (n = 65,340)	Std. Mean Difference
**Hemodynamic Data & Laboratory Values**			
PASYS	40.28 (10.76)	40.13 (10.78)	-0.013
PASYS missing (%)	33890 (51.87)	31454 (48.14)	-0.075
Ejection fraction	56.10 (11.36)	56.04 (11.36)	-0.005
EF missing (%)	3272 (5.01)	2374 (3.63)	-0.075
EF normal (%)	43236 (66.17)	43236 (66.17)	0
Creatinine	1.15 (0.91)	1.12 (0.85)	-0.022
Creatinine missing	434 (0.66)	172 (0.26)	-0.059
Albumin	3.81 (0.55)	3.83 (0.53)	0.036
Albumin missing (%)	13387 (20.49)	11857 (18.15)	-0.062
MELD score	9.13 (3.12)	8.97 (2.94)	-0.046
MELD score missing	17012 (26.04)	15427 (23.61)	-0.059
**Surgical Procedures**			
Previous CABG surgery (%)	3688 (5.64)	3070 (4.70)	-0.042
Previous valve surgery (%)	5314 (8.13)	4941 (7.56)	-0.020
IABP placed	2406 (3.68)	2019 (3.09)	-0.029
CathtoSurg	33.03 (52.96)	33.13 (48.90)	0.002
CathtoSurg missing	9275 (14.19)	7144 (10.93)	-0.098
CBP	130.62 (62.26)	127.59 (57.45)	-0.047
CBP missing	556 (0.85)	178 (0.27)	-0.091
Time in OR (min)	341.61 (114.99)	336.35 (107.51)	-0.044
Time in OR missing	238 (0.36)	182 (0.28)	-0.015
Time of skin incicion (min)	255.05 (102.37)	250.29 (96.26)	-0.045
Time of skin incicion missing	465 (0.71)	249 (0.38)	-0.044
Single valve (%)	62701 (95.96)	62701 (95.96)	0
**Surgery Type**			
AV repair (%)	621 (0.95)	621 (0.95)	0
AV replacement (%)	44638 (68.32)	44638 (68.32)	0
MV repair (%)	9412 (14.40)	9412 (14.40)	0
MV replacement (%)	8057 (12.33)	8057 (12.33)	0
Tricuspid valve repair/replacement (%)	2959 (4.53)	2959 (4.53)	0
Pulmonic valve repair/replacement (%)	219 (0.34)	219 (0.34)	0
Aortic root/valved conduit (Bentall) (%)	2637 (4.04)	2654 (4.06)	0.001
Aortic valve sparing root (%)	2214 (3.39)	2226 (3.41)	0.001
Aortic homograft or non-valved conduit (%)	964 (1.48)	947 (1.45)	-0.002
Plus CABG (%)	19998 (30.61)	19998 (30.61)	0
Plus other cardiac surgery (%)	13403 (20.51)	13403 (20.51)	0
**Predicted Mortality**			
Predicted Mortality Quartiles (%)			
None	18056 (27.63)	18056 (27.63)	0
1	7567 (11.58)	7567 (11.58)	0
2	10721 (16.41)	10721 (16.41)	0
3	14390 (22.02)	14390 (22.02)	0
4	14606 (22.35)	14606 (22.35)	0
Predicted Mortality, %	3.41 (4.27)	3.54 (4.57)	-0.007
**Valve Surgery Volume**			
Hospital-level	2592.69 (3692.46)	2592.69 (3692.46)	0
Surgeon-level	714.99 (572.94)	714.99 (572.94)	0

© 2022 MacKay EJ et al. *JAMA Network Open.*

6 eAppendix 6. Additional Details on Outcome Analysis

6.1 All-Patient Matched Comparison

6.1.1 Primary Outcome: 30-Day Mortality

We analyzed the binary outcome *30-day mortality* using McNemar's test (Cox and Snell, 1989; Rosenbaum, 2002). eTable 11 summarized the outcomes in McNemar format. Each count in eTable 11 represented a pair, not a patient. eTable 11 said that among 161, 610 matched pairs of two patients, one with TEE and the other without, neither patients died in 30 days in 147, 785 pairs, both died in 30 days in 849 pairs, the patient with TEE died in 30 days while the other survived in 30 days in 5, 309 pairs, and the patient without TEE died in 30 days while the other survived in 30 days in 7, 667 pairs. McNemar's test yielded a p-value $< 2.2 \times 10^{-16}$. The null hypothesis of no association between TEE monitoring and 30-day mortality was rejected at the 10^{-5} level.

eTable 11: 30-day mortality after valve surgery in 161,610 matched pairs of two patients, one with TEE and the other not. The table counts pairs, not patients.

		Without TEE		
		Dead	Alive	Total
	Dead	849	5,309	6,158
With TEE	Alive	7,667	147,785	155,452
	Total	8,516	153,094	161,610

6.1.2 Secondary Outcome I: 30-Day Mortality or Incidence of Stroke

eTable 12 summarized the outcomes in McNemar format. Among 161, 610 matched pairs of two patients, one with TEE and the other without, neither patients died or had a stroke in 30 days in 142, 597 pairs, both died or had a stroke in 30 days in 1, 306 pairs, the patient with TEE died or had a stroke in 30 days while the other neither died or had a stroke in 30 days in 7, 684 pairs, and the patient without TEE died or had a stroke in 30 days while the other neither died or had a stroke in 30 days in 10, 023 pairs. McNemar's test yielded a p-value $< 2.2 \times 10^{-16}$. The null hypothesis of no association between TEE monitoring and 30-day mortality or incidence of stroke was rejected at the 10^{-5} level.

eTable 12: 30-day mortality or incidence of stroke after valve surgery in 161,610 matched pairs of two patients, one with TEE and the other not. The table counts pairs, not patients.

		Without TEE		
		Dead	Alive	Total
	Dead	1,306	7,684	8,990
With TEE	Alive	10,023	142,597	152,620
	Total	11,329	150,281	161,610

6.1.3 Secondary Outcome II: 30-Day Mortality or Reoperation

eTable 13 summarized the outcomes in McNemar format. Among 161, 610 matched pairs of two patients, one with TEE and the other without, neither patients died or had a stroke in 30 days in 137, 423 pairs, both died or had a stroke in 30 days in 1, 744 pairs, the patient with TEE died or had a stroke in 30 days while the other neither died or had a stroke in 30 days in 9, 858 pairs, and the patient without TEE died or had a stroke in 30 days while the other neither died or had a stroke in 30 days in 12, 585 pairs. McNemar's test yielded a p-value $< 2.2 \times 10^{-16}$. The null hypothesis of no association between TEE monitoring and 30-day mortality or incidence of stroke was rejected at the 10^{-5} level.

eTable 13: 30-day mortality or incidence of reoperation after valve surgery in 161,610 matched pairs of two patients, one with TEE and the other not. The table counts pairs, not patients.

		Without TEE		
		Dead	Alive	Total
	Dead	1,744	9,858	11,602
With TEE	Alive	12,585	137,423	150,008
	Total	14,329	147,281	161,610

6.1.4 Negative Control Outcome: Post-Surgery Creatinine

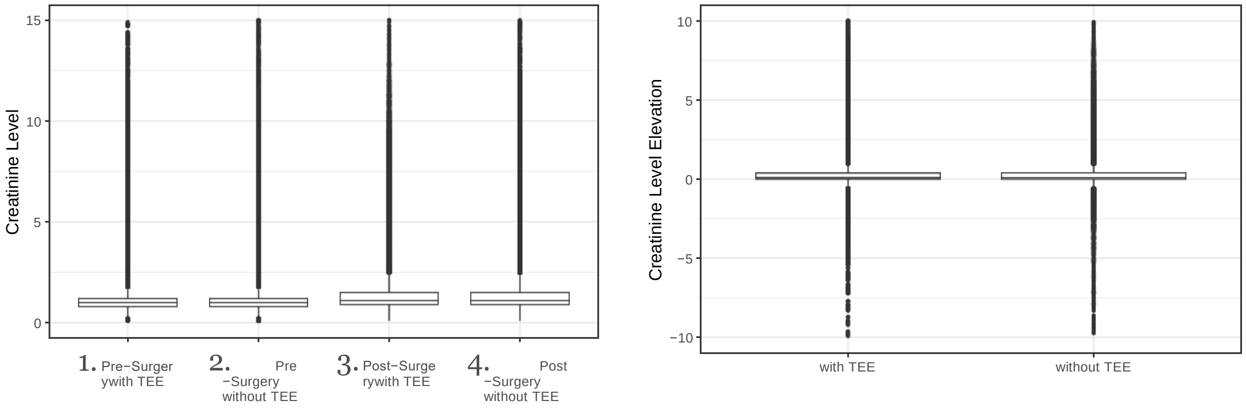

eFigure 5: Visualization of the pre-surgery and post-surgery creatinine level in the treated and control groups after matching. Left panel: boxplots of the pre-surgery and post-surgery creatinine in the matched treated and control groups. Right panel: elevation in the creatinine level (post-surgery minus pre-surgery) in the matched treated group versus matched control group.

The left panel of Figure 5 examines the pre-surgery and post-surgery creatinine level in the matched treated and matched control groups (excluding < 3.5% patients who did not have a post-surgery creatinine measurement). In the matched treated group, the creatinine level is 1.184 (pre-surgery) versus 1.492 (post-surgery); in the matched control group, the creatinine level is 1.173 (pre-surgery) versus 1.487 (post-surgery). On average, creatinine level elevates by $1.487 - 1.173 = 0.314$ in the with-TEE group and $1.492 - 1.184 = 0.308$ in the without-TEE group. We test the null hypothesis that TEE has no effect on the creatinine elevation (i.e., sample average treatment effect equal to 0). The p-value is 0.026 (95% CI : $[0.001, 0.012]$).

6.2 Within-Equivocal-Surgeon, Within-Hospital Matched Comparison

6.2.1 Primary Outcome: 30-Day Mortality

eTable 14 summarized the outcomes in McNemar format. Among 22, 739 matched pairs of two patients, one with TEE and the other without, neither patients died in 30 days in 21, 422 pairs, both died in 30 days in 49 pairs, the patient with TEE died in 30 days while the other survived in 30 days in 586 pairs, and the patient without TEE died in 30 days while the other survived in 30 days in 682 pairs. McNemar's test yielded a p-value = 0.008. The null hypothesis of no association between TEE monitoring and 30-day mortality was rejected at the 0.01 level.

eTable 14: 30-day mortality after valve surgery in 22,739 matched pairs of two patients, one with TEE and the other not. The table counts pairs, not patients.

		Without TEE		
		Dead	Alive	Total
With TEE	Dead	49	586	635
	Alive	682	21,422	22,104
	Total	731	22,008	22,739

6.2.2 Secondary Outcome I: 30-Day Mortality or Incidence of Stroke

eTable 15 summarized the outcomes in McNemar format. Among 22, 739 matched pairs of two patients, one with TEE and the other without, neither patients died or had a stroke in 30 days in 21, 656 pairs, both died or had a stroke in 30 days in 96 pairs, the patient with TEE died or had a stroke in 30 days while the other did not in 30 days in 900 pairs, and the patient without TEE died or had a stroke in 30 days while the other did not in 30 days in 987 pairs. McNemar's test yielded a p-value = 0.048. The null hypothesis of no association between TEE monitoring and 30-day mortality was rejected at the 0.05 level.

eTable 15: 30-day mortality or incidence of stroke after valve surgery in 22,739 matched pairs of two patients, one with TEE and the other not. The table counts pairs, not patients.

		Without TEE		
		Dead	Alive	Total
With TEE	Dead	96	900	996
	Alive	987	20,756	21,743
	Total	1,083	21,656	22,739

6.2.3 Secondary Outcome II: 30-Day Mortality or Reoperation

eTable 19 summarized the outcomes in McNemar format. Among 22, 739 matched pairs of two patients, one with TEE and the other without, neither patients died or had a stroke in 30 days in 20, 612 pairs, both died or had a stroke in 30 days in 665 pairs, the patient with TEE died or had a stroke in 30 days while the other neither died or had a stroke in 30 days in 708 pairs, and the patient without TEE died or had a stroke in 30 days while the other neither died or had a stroke in 30 days in 754 pairs. McNemar's test yielded a p-value 0.239. The null hypothesis of no association between TEE monitoring and 30-day mortality or incidence of stroke was not rejected at the 0.05 level.

eTable 16: 30-day mortality or incidence of reoperation after valve surgery in 22,739 matched pairs of two patients, one with TEE and the other not. The table counts pairs, not patients.

		Without TEE		
		Dead	Alive	Total
With TEE	Dead	665	708	1,373
	Alive	754	20,612	21,366
	Total	1,419	21,320	22,739

6.2.4 Negative Control Outcome: Post-Surgery Creatinine

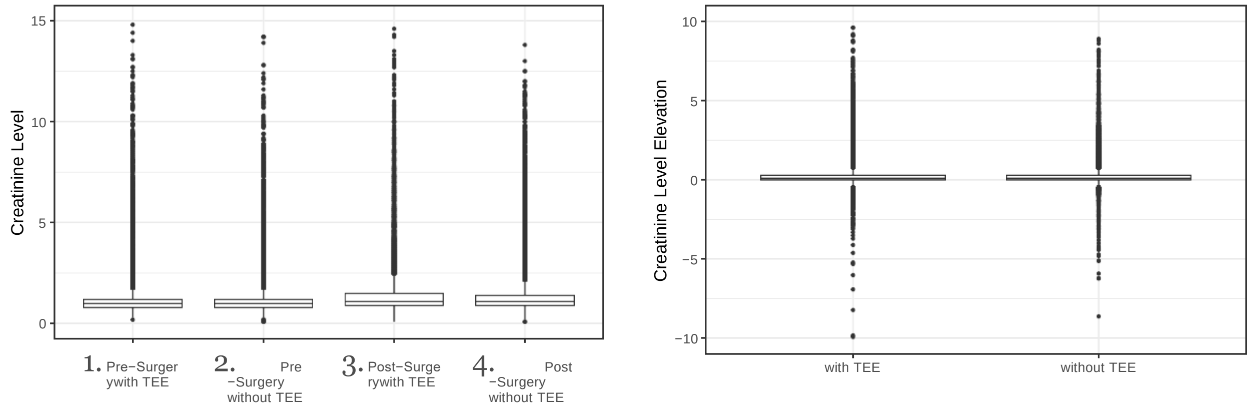

eFigure 6: Visualization of the pre-surgery and post-surgery creatinine level in the treated and control groups after matching. Left panel: boxplots of the pre-surgery and post-surgery creatinine in the matched treated and control groups. Right panel: elevation in the creatinine level (post-surgery minus pre-surgery) in the matched treated group versus matched control group.

The left panel of Figure 7 examines the pre-surgery and post-surgery creatinine level in the matched treated and matched control groups (excluding < 3% patients without a post-surgery creatinine measurement). In the matched treated group, the creatinine level is 1.131 (pre-surgery) versus 1.394 (post-surgery); in the matched control group, the creatinine level is 1.122 (pre-surgery) versus 1.389 (post-surgery). On average, creatinine level elevates by $1.394 - 1.131 = 0.263$ in the with-TEE group and 0.267 in the without-TEE group. We test the null hypothesis that TEE has no effect on the creatinine elevation (i.e., sample average treatment effect equal to 0). The p-value is 0.633 (95% CI : $[-0.016, 0.010]$.

6.3 Within-All-Surgeon, Within-Hospital Matched Comparison

6.3.1 Primary Outcome: 30-Day Mortality

eTable 17 summarized the outcomes in the McNemar format. Among 65, 340 matched pairs of two patients, one with TEE and the other without, neither patients died in 30 days in 60, 952 pairs, both died in 30 days in 169 pairs, the patient with TEE died in 30 days while the other survived in 30 days in 1, 760 pairs, and the patient without TEE died in 30 days while the other survived in 30 days in 2, 459 pairs. McNemar's test yielded a p-value < 0.0001. The null hypothesis of no association between TEE monitoring and 30-day mortality was rejected at the 10^{-5} level.

eTable 17: 30-day mortality after valve surgery in 65,340 matched pairs of two patients, one with TEE and the other not. The table counts pairs, not patients.

		Without TEE		
		Dead	Alive	Total
	Dead	169	1,760	1,929
With TEE	Alive	2,459	60,952	63,411
	Total	2,628	62,712	65,340

6.3.2 Secondary Outcome I: 30-Day Mortality or Incidence of Stroke

eTable 18 summarized the outcomes in McNemar format. Among 65, 340 matched pairs of two patients, one with TEE and the other without, neither patients died or had a stroke in 30 days in 61, 623 pairs, both died or had a stroke in 30 days in 3, 386 pairs, the patient with TEE died or had a stroke in 30 days while the other did not in 30 days in 2, 711 pairs, and the patient without TEE died or had a stroke in 30 days while the other did not in 30 days in 3, 386 pairs. McNemar's test yielded a p-value < 0.0001. The null hypothesis of no association between TEE monitoring and 30-day mortality was rejected at the 10^{-5} level.

eTable 18: 30-day mortality or incidence of stroke after valve surgery in 65,340 matched pairs of two patients, one with TEE and the other not. The table counts pairs, not patients.

		Without TEE		
		Dead	Alive	Total
	Dead	331	2,711	3,042
With TEE	Alive	3,386	58,912	62,298
	Total	3,717	61,623	65,340

6.3.3 Secondary Outcome II: 30-Day Mortality or Reoperation

eTable 19 summarized the outcomes in McNemar format. Among 65, 340 matched pairs of two patients, one with TEE and the other without, neither patients died or had a stroke in 30 days in 61, 010 pairs, both died or had a stroke in 30 days in 2, 028 pairs, the patient with TEE died or had a stroke in 30 days while the other neither died or had a stroke in 30 days in 1, 984 pairs, and the patient without TEE died or had a stroke in 30 days while the other neither died or had a stroke in 30 days in 2, 302 pairs. McNemar's test yielded a p-value < 0.0001. The null hypothesis of no association between TEE monitoring and 30-day mortality or incidence of stroke was rejected at the 10^{-5} level.

eTable 19: 30-day mortality or incidence of reoperation after valve surgery in 65,340 matched pairs of two patients, one with TEE and the other not. The table counts pairs, not patients.

		Without TEE		
		Dead	Alive	Total
	Dead	2,028	1,984	4,012
With TEE	Alive	2,302	59,026	61,328
	Total	4,330	61,010	65,340

6.3.4 Negative Control Outcome: Post-Surgery Creatinine

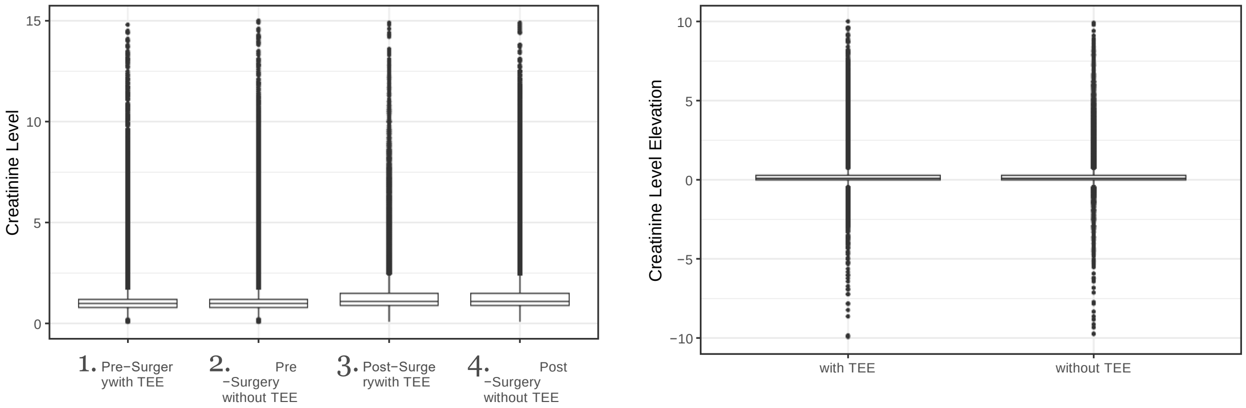

eFigure 7: Visualization of the pre-surgery and post-surgery creatinine level in the treated and control groups after matching. Left panel: boxplots of the pre-surgery and post-surgery creatinine in the matched treated and control groups. Right panel: elevation in the creatinine level (post-surgery minus pre-surgery) in the matched treated group versus matched control group.

The left panel of Figure 7 examines the pre-surgery and post-surgery creatinine level in the matched treated and matched control groups (excluding < 3% patients without a post-surgery creatinine measurement). In the matched treated group, the creatinine level is 1.122 (pre-surgery) versus 1.392 (post-surgery); in the matched control group, the creatinine level is 1.144 (pre-surgery) versus 1.426 (post-surgery). On average, creatinine level elevates by $1.392 - 1.122 = 0.270$ in the with-TEE group and 0.282 in the without-TEE group. We test the null hypothesis that TEE has no effect on the creatinine elevation (i.e., sample average treatment effect equal to 0), and the p-value is 0.002 (95% CI : $[-0.021, -0.005]$). We detected a very minor effect of TEE on creatinine elevation.

6.4 Summary

eTable 20 summarizes results of the unadjusted analysis and three matched comparisons.

eTable 20: Comparison of Outcomes Between Matched Patients Undergoing Valve Surgery with TEE and without TEE

**Unadjusted Analysis**	TEE (n = 711,326)	no TEE (n = 161,610)	Odds Ratio (95% CI)	Difference (95% CI)	*p* value
30-day mortality	3.92%	5.27%	0.73 (0.72 to 0.75)	NA	< 0.0001
30-day mortality or stroke	5.63%	7.01%	0.79 (0.77 to 0.81)	NA	< 0.0001
30-day mortality or reoperation	7.31%	8.87%	0.81 (0.79 to 0.83)	NA	< 0.0001
Creatinine elevation	0.317	0.309	NA	0.008 (0.003 to 0.012)	0.002
**All-Patient Match**	TEE (n = 161,610)	no TEE (n = 161,610)	Odds Ratio (95% CI)	Difference (95% CI)	*p* value
30-day mortality	3.81%	5.27%	0.69 (0.67 to 0.72)	NA	< 0.0001
30-day mortality or stroke	5.56%	7.01%	0.77 (0.74 to 0.79)	NA	< 0.0001
30-day mortality or reoperation	7.18%	8.87%	0.78 (0.76 to 0.80)	NA	< 0.0001
Creatinine elevation	0.314	0.308	NA	0.007 (0.001 to 0.012)	0.028
**Within Equivocal Surgeons**	TEE (n = 22,739)	no TEE (n = 22,739)	Odds Ratio (95% CI)	Difference (95% CI)	*p* value
30-day mortality	2.79%	3.22%	0.86 (0.77 to 0.96)	NA	0.008
30-day mortality or stroke	4.38%	4.76%	0.91 (0.83 to 1.00)	NA	0.048
30-day mortality or reoperation	6.04%	6.24%	0.94 (0.85 to 1.04)	NA	0.239
Creatinine elevation	0.263	0.267	NA	-0.003 (-0.016 to 0.010)	0.633
**Within All Surgeons**	TEE (n = 65,340)	no TEE (n = 65,340)	Odds Ratio (95% CI)	Difference (95% CI)	*p* value
30-day mortality	2.95%	4.02%	0.72 (0.67 to 0.76)	NA	< 0.0001
30-day mortality or stroke	4.66%	5.69%	0.80 (0.76 to 0.84)	NA	< 0.0001
30-day mortality or reoperation	6.14%	6.63%	0.86 (0.81 to 0.92)	NA	< 0.0001
Creatinine elevation	0.270	0.282	NA	-0.013 (-0.021 to -0.005)	0.002

7 eAppendix 7. Subgroup analysis

7.1 Defining subgroups

We considered a subgroup analysis facilitated by the exact matching on the surgery type in the two within-surgeon, within-hospital matched comparisons. We considered the following six subgroups.

Subgroup I AV replacement only

Subgroup II AV replacement + CABG **OR** AV replacement + CABG + other **OR** AV replacement + other

Subgroup III MV replacement only **OR** MV replacement + CABG **OR** MV replacement + other **OR** AV replacement + MV replacement

Subgroup IV MV repair only **OR** MV repair + CABG **OR** MV repair + other

Subgroup V Aortic proximal only **OR** Aortic proximal + other

Subgroup VI Not belonging to Subgroup I-V

eTable 21: Within-All-Surgeon, Within-Hospital Subgroup Analysis Results

**Subgroup I**	TEE (n = 22,656)	no TEE (n = 22,656)	Odds Ratio (95% CI)	*p* value
30-day mortality	1.71%	2.17%	0.78 (0.68 to 0.89)	0.0003
30-day mortality or stroke	2.93%	3.35%	0.86 (0.77 to 0.96)	0.009
30-day mortality or reoperation	4.29%	4.76%	0.84 (0.75 to 0.94)	< 0.0001
**Subgroup II**	TEE (n = 18,553)	no TEE (n = 18,553)	Odds Ratio (95% CI)	*p* value
30-day mortality	3.21%	4.09%	0.77 (0.69 to 0.86)	< 0.0001
30-day mortality or stroke	5.07%	5.95%	0.84 (0.77 to 0.92)	0.0002
30-day mortality or reoperation	6.55%	7.12%	0.85 (0.76 to 0.95)	0.0039
**Subgroup III**	TEE (n = 6,208)	no TEE (n = 6,208)	Odds Ratio (95% CI)	*p* value
30-day mortality	5.04%	6.96%	0.71 (0.60 to 0.82)	< 0.0001
30-day mortality or stroke	6.98%	8.72%	0.78 (0.68 to 0.89)	0.0003
30-day mortality or reoperation	9.13%	9.83%	0.85 (0.71 to 1.01)	0.066
**Subgroup IV**	TEE (n = 7,066)	no TEE (n = 7,066)	Odds Ratio (95% CI)	*p* value
30-day mortality	1.54%	1.73%	0.89 (0.67 to 1.17)	0.418
30-day mortality or stroke	2.87%	2.72%	1.06 (0.86 to 1.31)	0.606
30-day mortality or reoperation	3.74%	3.98%	0.90 (0.71 to 1.13)	0.367
**Subgroup V**	TEE (n = 3,371)	no TEE (n = 3,371)	Odds Ratio (95% CI)	*p* value
30-day mortality	4.36%	7.27%	0.58 (0.46 to 0.72)	< 0.0001
30-day mortality or stroke	7.57%	11.33%	0.62 (0.52 to 0.74)	< 0.0001
30-day mortality or reoperation	8.69%	9.91%	0.77 (0.61 to 0.97)	0.024
**Subgroup VI**	TEE (n = 7,486)	no TEE (n = 7,486)	Odds Ratio (95% CI)	*p* value
30-day mortality	5.04%	7.73%	0.61 (0.53 to 0.71)	< 0.0001
30-day mortality or stroke	7.31%	9.89%	0.70 (0.62 to 0.79)	< 0.0001
30-day mortality or reoperation	9.35%	9.42%	0.98 (0.84 to 1.16)	0.871

eTable 22: Within-Equivocal-Surgeon, Within-Hospital Subgroup Analysis Results

**Subgroup I**	TEE (n = 8,434)	no TEE (n = 8,434)	Odds Ratio (95% CI)	*p* value
30-day mortality	1.51%	1.93%	0.77 (0.60 to 0.98)	0.034
30-day mortality or stroke	2.69%	3.08%	0.86 (0.72 to 1.04)	0.133
30-day mortality or reoperation	4.35%	4.55%	0.93 (0.78 to 1.12)	0.468
**Subgroup II**	TEE (n = 6,698)	no TEE (n = 6,698)	Odds Ratio (95% CI)	*p* value
30-day mortality	3.39%	3.33%	1.02 (0.84 to 1.24)	0.885
30-day mortality or stroke	5.12%	4.96%	1.04 (0.88 to 1.21)	0.690
30-day mortality or reoperation	6.85%	6.78%	1.02 (0.84 to 1.24)	0.847
**Subgroup III**	TEE (n = 2,290)	no TEE (n = 2,290)	Odds Ratio (95% CI)	*p* value
30-day mortality	5.11%	6.07%	0.83 (0.63 to 1.08)	0.168
30-day mortality or stroke	6.73%	7.86%	0.84 (0.66 to 1.06)	0.143
30-day mortality or reoperation	9.04%	9.78%	0.84 (0.63 to 1.12)	0.252
**Subgroup IV**	TEE (n = 2,231)	no TEE (n = 2,231)	Odds Ratio (95% CI)	*p* value
30-day mortality	1.26%	1.39%	0.89 (0.50 to 1.59)	0.784
30-day mortality or stroke	2.29%	2.29%	1.00 (0.65 to 1.54)	1
30-day mortality or reoperation	3.45%	3.86%	0.84 (0.56 to 1.26)	0.435
**Subgroup V**	TEE (n = 834)	no TEE (n = 834)	Odds Ratio (95% CI)	*p* value
30-day mortality	4.80%	5.52%	0.86 (0.54 to 1.37)	0.576
30-day mortality or stroke	7.31%	9.23%	0.77 (0.53 to 1.11)	0.174
30-day mortality or reoperation	9.11%	9.35%	0.95 (0.58 to 1.54)	0.908
**Subgroup VI**	TEE (n = 2,252)	no TEE (n = 2,252)	Odds Ratio (95% CI)	*p* value
30-day mortality	4.26%	5.73%	0.72 (0.54 to 0.96)	0.024
30-day mortality or stroke	7.11%	8.13%	0.86 (0.68 to 1.08)	0.203
30-day mortality or reoperation	8.30%	8.57%	0.93 (0.68 to 1.27)	0.705

8 eAppendix 8. Sensitivity Analysis I: Assessing Robustness of Primary OutcomeFindings to Unmeasured Confounding

In a sensitivity analysis, we investigate how large a bias from unmeasured confounding could alter our primary analysis results. We used a methodology developed in Rosenbaum (2002, Section 4.3.1) and Rosenbaum and Silber (2009) and implemented via R package rbounds and sensitivitymv (Rosenbaum, 2015). The conventional two-sided *p*-value from McNemar's test for our primary outcome 30-day mortality in the all-patient matched comparison was $< 2.2 \times 10^{-16}$, but it could increase to approximately 0.05 had there existed an unmeasured confounder that *doubled* the odds of death and simultaneously *tripled* the odds of receiving intraoperative TEE. Similarly, the conventional two-sided *p*-value from McNemar's test for our primary outcome 30-day mortality in the within-surgeon, within-hospital matched comparison was 0.008, but it could increase to approximately 0.05 had there existed an unmeasured confounder that increased the odds of death by 40% and simultaneously increased the odds of receiving intraoperative TEE by 40%.

9 eAppendix 9. Sensitivity Analysis II: Assessing Robustness of Results to theMissingness of the TEE Status

eTable 23: Comparison of Outcomes in a Sensitivity Analysis where Subjects with Missing TEE Status were Excluded.

**Unadjusted Analysis**	TEE (n = 711,326)	no TEE (n = 142,995)	Odds Ratio (95% CI)	Difference (95% CI)	*p* value
30-day mortality	3.92%	5.44%	0.71 (0.69 to 0.73)	NA	< 0.0001
30-day mortality or stroke	5.63%	7.20%	0.77 (0.75 to 0.79)	NA	< 0.0001
30-day mortality or reoperation	7.31%	9.05%	0.79 (0.78 to 0.81)	NA	< 0.0001
Creatinine elevation	0.317	0.311	NA	0.005 (0.000 to 0.010)	0.049
**All-Patient Match**	TEE (n = 142,995)	no TEE (n = 142,995)	Odds Ratio (95% CI)	Difference (95% CI)	*p* value
30-day mortality	3.84%	5.44%	0.67 (0.65 to 0.70)	NA	< 0.0001
30-day mortality or stroke	5.58%	7.20%	0.74 (0.72 to 0.77)	NA	< 0.0001
30-day mortality or reoperation	7.23%	9.05%	0.77 (0.75 to 0.79)	NA	< 0.0001
Creatinine elevation	0.317	0.311	NA	0.006 (0.000 to 0.012)	0.050
**Within, Equivocal Surgeons**	TEE (n = 18,913)	no TEE (n = 18,913)	Odds Ratio (95% CI)	Difference (95% CI)	*p* value
30-day mortality	2.92%	3.39%	0.85 (0.75 to 0.96)	NA	0.008
30-day mortality or stroke	4.56%	4.94%	0.91 (0.83 to 1.01)	NA	0.075
30-day mortality or reoperation	6.17%	6.27%	0.97 (0.86 to 1.08)	NA	0.239
Creatinine elevation	0.270	0.273	NA	-0.002 (-0.016 to 0.012)	0.755
**Within, All Surgeons**	TEE (n = 58,946)	no TEE (n = 58,946)	Odds Ratio (95% CI)	Difference (95% CI)	*p* value
30-day mortality	3.03%	4.17%	0.71 (0.66 to 0.76)	NA	< 0.0001
30-day mortality or stroke	4.76%	5.85%	0.80 (0.75 to 0.84)	NA	< 0.0001
30-day mortality or reoperation	6.23%	6.73%	0.86 (0.81 to 0.92)	NA	< 0.0001
Creatinine elevation	0.274	0.288	NA	-0.014 (-0.023 to -0.006)	0.001

10 eAppendix 10. Details On the Negative Control Outcome

10.1 Rationale Behind Negative Control Outcome Selection

While all adjusted analyses agreed and all held up against robust statistical sensitivity analyses, we felt a negative control outcome analysis could serve as an additional check for the validity of our findings associating TEE to improved outcomes. An ideal negative control outcome is a factor that would be implausibly related to TEE use (or lack of use). We considered multiple possibilities for a negative control outcome including: surgical site infection, deep vein thrombosis, and arrhythmia. But while all of these outcomes were appropriate (e.g. implausibly related to TEE use) all were associated with an increased rate of mortality and suffered from survival bias. Therefore, we felt none of these could be used as a negative control unless combined with mortality and analyzed as a composite, which was not suitable given our primary outcome was mortality. But, a postoperative creatinine level was obtained in every patient (even those patients who ultimately died). Therefore, we selected creatinine elevation – defined as postoperative creatinine level minus preoperative creatinine level – as our negative control outcome. Elevation in creatinine level is an ideal negative control outcome to test for residual confounding comparing TEE vs no TEE for the following three reasons.

1. In our cohort, postoperative creatinine was a laboratory value obtained in every patient (even those who ultimately died) immediately postoperatively and free from survival bias.

2. While creatinine elevation is highly probable after cardiac surgery (e.g. increased after surgery compared to pre-surgery), it is reasonable to assume that the increase in creatinine would be roughly evenly allocated across both the TEE and no TEE groups.

3. Even if the above assumption (point 2) was untrue, and the TEE-guided optimization of hemodynamic management during cardiac surgery limited the degree of acute kidney injury, the TEE group would have a smaller elevation in creatinine (compared to the non-TEE group). If the TEE group demonstrated a statistically significantly greater elevation in creatinine (compared to the no TEE group) this "worse outcome" result of the negative control outcome analysis would be in disagreement with the "improved outcome" results of the primary analyses. In other words, a statistically significant finding of a greater elevation in creatinine in the TEE group would represent a bias against our primary finding of improved outcome with TEE – indicating our primary findings of an association between TEE and improved outcomes could be an underestimate of the true clinical outcomes benefit.

10.2 Comment on Results of Negative Control

As can be seen in eTable 20, TEE was associated with a statistically significantly greater increase creatinine elevation (e.g. "worse outcome") on the unadjusted analysis: (0.317 TEE vs 0.309 no TEE; p¡0.002); and the adjusted, all-patient matched analysis (0.314 TEE vs 0.308 no TEE; p=0.028). On the within-hospital, within-equivocal surgeon matched analysis, there was no statistically significant difference in creatinine elevation among the TEE group (vs no TEE group) (0.263 vs 0.267; 0.633). Only on the within-hospital, within-all surgeon matched analysis did the TEE group demonstrate a statistically significantly smaller increase in creatinine elevation compared to the no TEE group (0.270 vs 0.282; p=0.002). Three of the four negative control outcome analyses are either statistically insignificant or incongruent with the primary results. Compellingly, the incongruent results

of the TEE group demonstrating a greater increase in postoperative creatinine indicates the possibility of residual bias against our finding that TEE is associated with improved clinical outcomes. The only result that indicates the possibility of residual confounding is the within-all-surgeon match where the TEE group demonstrates a statistically significant smaller increase (e.g. “better outcome”) compared to the no TEE group. Admittedly, this does indicate the possibility that including the high-TEE-surgeons into the within-hospital, within-surgeon match (as opposed to the analysis including only the equivocal-TEE-surgeons) reintroduced confounding between TEE and outcomes. But while statistically significant differences were observed in creatinine elevation between the TEE and no TEE groups – all differences were between -0.013 and +0.007 and not considered to be a clinically meaningful difference in creatinine between the TEE and no TEE groups.

11 eAppendix 11. R and Stata Code

All analyses including data preprocessing, statistical matching, and outcome analyses can be reproduced using the R and Stata code stored in the following GitHub code repository: https://github.com/emily-jane-mackay/Echocardiography_STS

eReferences

Cox, D. R. and Snell, E. J. (1989). *Analysis of binary data*, volume 32. CRC press.

Friedman, J., Hastie, T., and Tibshirani, R. (2010). Regularization paths for generalized linear models via coordinate descent. *Journal of statistical software*, 33(1):1.

Mahalanobis, P. C. (1936). On the generalized distance in statistics. National Institute of Science of India.

Rosenbaum, P. R. (1989). Optimal matching for observational studies. *Journal of the American Statistical Association*, 84(408):1024–1032.

Rosenbaum, P. R. (2002). *Observational Studies*. Springer.

Rosenbaum, P. R. (2010). *Design of observational studies*. Springer.

Rosenbaum, P. R. (2012). Optimal matching of an optimally chosen subset in observational studies. *Journal of Computational and Graphical Statistics*, 21(1):57–71.

Rosenbaum, P. R. (2015). Two r packages for sensitivity analysis in observational studies. *Observational Studies*, 1(1):1–17.

Rosenbaum, P. R. (2020). Modern algorithms for matching in observational studies. *Annual Review of Statistics and Its Application*, 7:143–176.

Rosenbaum, P. R., Ross, R. N., and Silber, J. H. (2007). Minimum distance matched sampling with fine balance in an observational study of treatment for ovarian cancer. *Journal of the American Statistical Association*, 102(477):75–83.

Rosenbaum, P. R. and Rubin, D. B. (1983). The central role of the propensity score in observational studies for causal effects. *Biometrika*, 70(1):41–55.

Rosenbaum, P. R. and Rubin, D. B. (1985). Constructing a control group using multivariate matched sampling methods that incorporate the propensity score. *The American Statistician*, 39(1):33–38.

Rosenbaum, P. R. and Silber, J. H. (2009). Amplification of sensitivity analysis in matched observational studies. *Journal of the American Statistical Association*, 104(488):1398–1405.

Rubin, D. B. (1980). Bias reduction using mahalanobis-metric matching. *Biometrics*, pages 293–298.

Yu, R., Silber, J. H., Rosenbaum, P. R., et al. (2020). Matching methods for observational studies derived from large administrative databases. *Statistical Science*, 35(3):338–355.
